# SPONGEdb: a pan-cancer resource for competing endogenous RNA interactions

**DOI:** 10.1093/narcan/zcaa042

**Published:** 2021-01-06

**Authors:** Markus Hoffmann, Elisabeth Pachl, Michael Hartung, Veronika Stiegler, Jan Baumbach, Marcel H Schulz, Markus List

**Affiliations:** Chair of Experimental Bioinformatics, TUM School of Life Sciences, Technical University of Munich, 85354 Freising, Germany; Chair of Experimental Bioinformatics, TUM School of Life Sciences, Technical University of Munich, 85354 Freising, Germany; Chair of Experimental Bioinformatics, TUM School of Life Sciences, Technical University of Munich, 85354 Freising, Germany; Chair of Experimental Bioinformatics, TUM School of Life Sciences, Technical University of Munich, 85354 Freising, Germany; Chair of Experimental Bioinformatics, TUM School of Life Sciences, Technical University of Munich, 85354 Freising, Germany; Institute for Cardiovascular Regeneration, Goethe University, 60596 Frankfurt am Main, Germany; German Center for Cardiovascular Research, Partner site Rhein-Main, 60590 Frankfurt am Main, Germany; Cardio-Pulmonary Institute, Goethe University Hospital, 60596 Frankfurt am Main, Germany; Chair of Experimental Bioinformatics, TUM School of Life Sciences, Technical University of Munich, 85354 Freising, Germany

## Abstract

microRNAs (miRNAs) are post-transcriptional regulators involved in many biological processes and human diseases, including cancer. The majority of transcripts compete over a limited pool of miRNAs, giving rise to a complex network of competing endogenous RNA (ceRNA) interactions. Currently, gene-regulatory networks focus mostly on transcription factor-mediated regulation, and dedicated efforts for charting ceRNA regulatory networks are scarce. Recently, it became possible to infer ceRNA interactions genome-wide from matched gene and miRNA expression data. Here, we inferred ceRNA regulatory networks for 22 cancer types and a pan-cancer ceRNA network based on data from The Cancer Genome Atlas. To make these networks accessible to the biomedical community, we present SPONGEdb, a database offering a user-friendly web interface to browse and visualize ceRNA interactions and an application programming interface accessible by accompanying R and Python packages. SPONGEdb allows researchers to identify potent ceRNA regulators via network centrality measures and to assess their potential as cancer biomarkers through survival, cancer hallmark and gene set enrichment analysis. In summary, SPONGEdb is a feature-rich web resource supporting the community in studying ceRNA regulation within and across cancer types.

## INTRODUCTION

microRNAs (miRNAs) are important non-coding, post-transcriptional regulators that are involved in many biological processes and human diseases (1). miRNAs regulate their target RNA transcripts by either degrading them or by preventing their translation (2), thus acting as rheostats that regulate gene expression and maintain the functional balance of gene networks (3). The competing endogenous RNA (ceRNA) hypothesis suggests that RNA transcripts sharing binding sites for the same miRNA are in competition (4). Hence, miRNA–ceRNA interactions follow a many-to-many relationship where one miRNA can affect multiple ceRNA targets, and one ceRNA can contain multiple binding sites for various miRNAs (5), leading to complex cross-talk.

Since failures of these complex regulatory systems may lead to cancer (6), it is crucial to infer and compare gene-regulatory networks across various cancer types. However, previous network inference efforts focused mostly on transcription factor regulation (7–9), neglecting the influence of miRNA regulation and ceRNA competition. To close this gap, several efforts have been directed at developing methods for ceRNA network inference (10), which were applied to individual cancer types (11) but also across cancer types (12).

A number of resources have been proposed for studying ceRNAs, including miRTissue_*ce*_ (13), LnCeVar (14), Pan-ceRNADB (15), miRTarBase (16), ceRDB (17), lnCeDB (18), miRSponge (19), LncACTdb (20), miRcode (21), and starBase v2.0 (22) (see Supplementary Section S7 and Tables S1–3 for a systematic comparison). Only a few of these databases are dedicated to ceRNA networks. Pan-ceRNADB covers 20 cancer types (15) and considers messenger RNAs (mRNAs) in addition to lncRNAs as potential ceRNAs. In contrast, the LnCeVar database focuses on lncRNAs with experimental evidence for ceRNA interactions and adds additional analyses on the effect of single nucleotide and copy number variants on ceRNA activity (14). Existing resources currently do not afford genome-wide coverage, i.e. are limited to lncRNAs, or employ simplistic methods, which lead to a large number of false positives. For instance, only considering if two genes are enriched for shared miRNA regulation in a hypergeometric test neglects that both genes and the miRNA have to be co-expressed. Examining the correlation of gene expression with a shared miRNA does not quantify the effect on gene–gene correlation.

This highlights the need for a comprehensive and easily accessible resource of pan-cancer ceRNA networks that leverage state-of-the-art methods that account for the triangle relationship of ceRNA–miRNA–ceRNA triplets. Since methods leveraging conditional mutual information such as CUPID (23) or JAMI (24) do not afford genome-wide coverage due to computational costs, we have previously developed the method SPONGE, which overcomes limitations of sensitivity correlation (25) and facilitates fast genome-wide ceRNA network inference (26).

**Figure 1.**
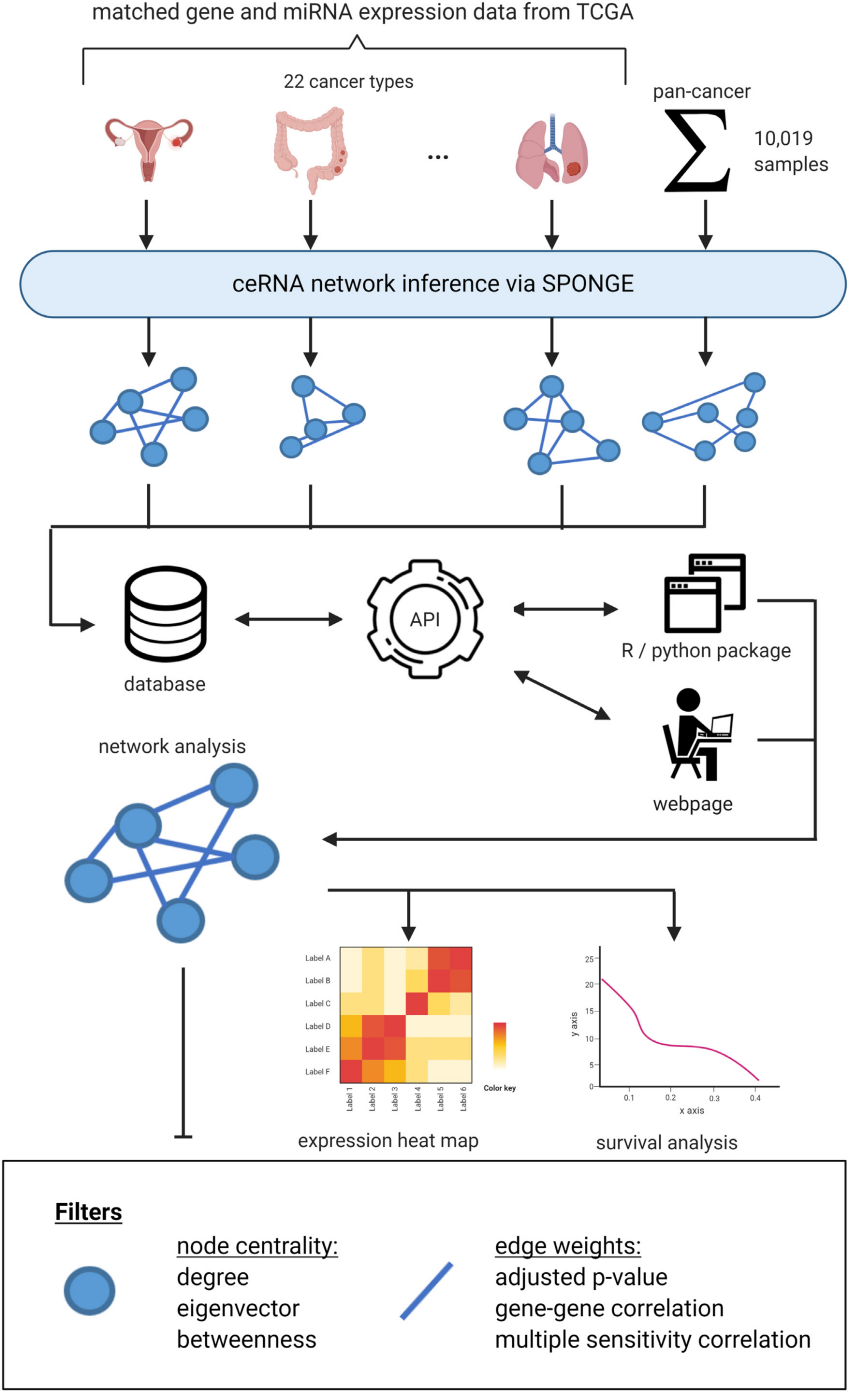
SPONGEdb overview: ceRNA networks for 22 cancer types as well as a pan-cancer ceRNA network for all 10 019 samples in TCGA have been inferred with SPONGE (26) and can be queried efficiently via a RESTful API through any programming language. We provide R and Python packages to simplify scripted access and offer a user-friendly and feature-rich web interface offering additional insights into ceRNA from external resources (ENCODE (34) for additional gene information, miRBase for additional miRNA information, Quick GO for correlating Gene Ontologies and the WikiPathways keys for each gene). The web interface offers visualization of the ceRNA networks, expression heat maps and survival analysis via Kaplan–Meier plots. Various filters for node centrality and edge weights allow refining the ceRNA network to focus on cancer-type-specific essential ceRNA regulators and their strongest or most significant interactions.

Here, we present SPONGEdb, a database providing access to SPONGE-inferred genome-wide ceRNA networks of 22 cancer types in TCGA (27) and a pan-cancer ceRNA network considering all available data (Figure 1). In contrast to most other resources, SPONGEdb results account for the contribution of multiple miRNAs and address previously neglected confounding factors. Specifically, we use the SPONGE R package (26) to infer empirical *P*-values that limit the false positive rate and avoid spurious correlations. SPONGEdb offers additional insights into the results through providing user-friendly features for accessibility not found in other databases: (i) SPONGEdb reported ceRNA interactions are easily accessible via a well-documented application programming interface (API); (ii) SPONGEdb allows investigating the effect of individual or multiple ceRNAs on a global ceRNA network, highlighting their relevance in a cancer background; a user-friendly web interface allows for searching and filtering ceRNA interactions and to visualize them as an interactive network enriched with additional information about patient survival as well as gene expression or functional annotations from WikiPathways (28), GeneCards (29), Quick GO (30), and Cancer Hallmarks (31). Functional enrichment analysis can be performed via g:Profiler (32). In addition to the web interface, we provide R and Python packages to allow third-party developers, data scientists, and biomedical researchers to carry out in-depth analyses. Here, we give an overview of the features of SPONGEdb and present a series of use cases demonstrating how this unique resource can be used for explorative analysis and hypothesis generation when studying ceRNA competition in cancer. Furthermore, we present results of SPONGE with experimentally validated data retrieved from Tay *et al.* (33) and miRSponge (19) using the features of SPONGEdb.

## MATERIALS AND METHODS

### Data processing

Using the SPONGE (26) bioconductor package, we identified significant ceRNA interactions for 10 019 samples for which paired gene, and miRNA expression data were available in TCGA (27). Data were processed by the TOIL project (35) and downloaded from the TOIL data hub in the Xena browser (36). Expression values were log2 transformed, and genes and miRNAs were discarded if not expressed in 80% or more of the samples or when variance was <0.5. To cover both non-coding and coding miRNA interactions, we used sequence-based predictions of several methods, namely TargetScan 7.1 (37) and miRcode v.11 (21). We further consider experimental evidence reported by miRTarBase 7.0 (38,39) and DIANA-LncBase v2 (40,41). SPONGE infers a ceRNA interaction network from the paired gene and miRNA expression data. SPONGE was applied to 22 cancer types in TCGA with >100 samples to obtain robust results for the correlations as well as for the aggregated pan-cancer dataset using default settings. For more details, we refer to the original method publication (26). Survival information from TCGA was used to produce Kaplan-Meier plots, where patients were split into two groups based on the median expression level. We used a log-rank test for assessing if the two survival curves differ significantly.

### Implementation

#### Centrality measures

Centrality measures provide important information about the organization of complex systems in network analysis (42,43). Del Rio *et al.* showed that a combination of at least two centrality measures achieve reliable performance in biomarker detection (44). Hence, we provide degree, betweenness and eigenvector centrality (45) in SPONGEdb.

#### Degree centrality

The degree centrality corresponds to the number of edges connected to a node and highlights hub nodes (42), which are known to be important in biological systems (46–48).

#### Betweenness centrality

Here, we consider bottlenecks rather than hubs of a network to be important, i.e. betweenness centrality $c_i^{BC}(g)$ of node *i* in a graph *g* is high if *i* falls on a large fraction of shortest paths between other nodes *j* and *k* (49). Formally,

(1) $$\begin{equation*} c_i^{BC}(g) = \sum \limits _{(j,k),j\not=i,j=i} \dfrac{v_g(i:j,k)}{v_g(j,k)} \end{equation*}$$

with $v$_*g*_(*i*: *j*, *k*), the number of shortest paths between *j* and *k* that visit *i* and $v$_*g*_(*j*, *k*) the number of all shortest paths between *j* and *k*. Raman et al. demonstrated that betweenness centrality correlate with lethality of organisms in protein networks (50).

#### Eigenvector centrality

Here, the importance of a node *i* is related to the importance of neighbors *j* connected by edge *a*_*ij*_, i.e. eigenvector centrality $c_i^{EC}$ is defined as:

(2) $$\begin{equation*} c_i^{EC} =\frac{1}{\lambda }\sum \limits _{j=1}^na_{ij}c_j^{EC} \end{equation*}$$

where *a*_*ij*_ is 1 if *i* and *j* are connected and else 0. λ corresponds to the eigenvalue which is unique under the requirement that all values of the eigenvector are positive. Negre *et al.* showed the importance of eigenvector centrality for biological pathways by creating a protein allosteric pathway network (51).

The SPONGEdb database contains expression and survival data from TCGA, gene anotations from ENCODE (34) and miRNA annotations from miRBase (52). For more details about the implementation of SPONGEdb, see Supplementary Sections S1–4 and Figures S1 and 2.

## RESULTS AND DISCUSSION

SPONGEdb allows users to query ceRNA interactions for 22 cancer types, as well as a pan-cancer ceRNA network via an API for which we implement three use cases: (i) a user-friendly web interface, (ii) an R package, and (iii) a Python package. The general architecture and work-flow of SPONGEdb are shown in Figure 1. In the following, we present three application cases that illustrate the broad utility of SPONGEdb in ceRNA research.

### Pan-cancer analysis of ceRNA activity

**Figure 2.**
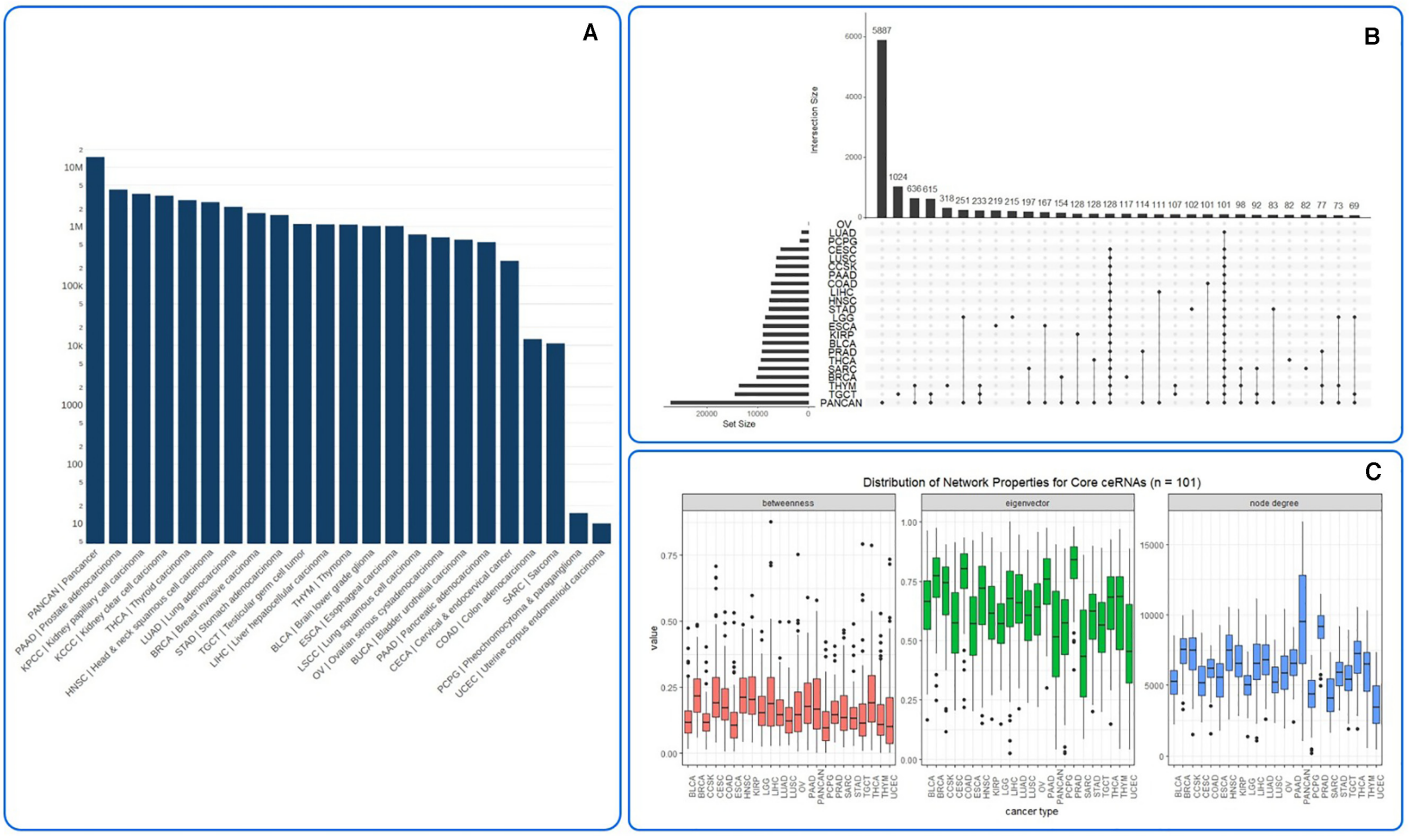
(**A**) Number of significant ceRNA interactions for each cancer type on a logarithmic scale. (**B**) Many ceRNAs are specific for a certain cancer type. Only two subsets of core ceRNA interactions are active in almost all cancer types (subsets with 101 and 128 ceRNAs, respectively). (**C**) Distribution of network properties for core ceRNAs for the subset of 101 ceRNA interactions, highlighting that core ceRNAs have only moderate regulatory strength.

SPONGEdb reports many significant interactions across 20 of the 22 cancer types as well as a unique pan-cancer analysis revealing key ceRNAs that play a role across cancer types (Figure 2A). Many of the candidate ceRNAs implicated here have not been described in the literature and can thus serve to prioritize candidates for experimental validation. Only a fraction of ceRNAs appear to act across all cancer types and, conversely, many ceRNAs act in a cancer-specific fashion (Figure 2B and C). As expected, we observe a positive trend between the number of tumor samples (e.g. breast cancer has 1063 samples, while thymoma has only 119 samples) and the number of significant interactions (Figure 4), which can be explained by an increase in statistical power. Fitting this hypothesis, the pan-cancer analysis comprising all cohorts provides the largest number of significant interactions. However, for some cancer types, we find only very few significant interactions, even if the sample numbers would suggest otherwise. Likewise, we observe a difference in the number of significant interactions for cancer types with similar cohort sizes.

Ovarian serous cystadeno carcinoma and uterine corpus endometrioid carcinoma appear as outliers with <1% of significant interactions (Figure 2), suggesting that in these cancer types, ceRNA regulation may either be less pronounced than in other cancer types or confounded by heterogeneity, i.e. introduced by the tumor microenvironment or by subtype-specific differences. To further investigate this, we performed SPONGE analysis on an independent Australian cohort of ovarian cancer patients (https://dcc.icgc.org/releases/current/Projects/OV-AU) where we also found <1% of significant interactions (see Supplementary Section S8 and Table S4), confirming the results on TCGA data.

There are other factors to consider that may limit the sensitivity of SPONGE, e.g. the high number of significant ceRNA interactions in testicular germ cell tumors could be explained by a relatively high complexity of the transcriptome in this tissue (53). Moreover, we consider only gene–miRNA interactions with a negative regression coefficient and may thus miss cases where translational repression rather than degradation of the target transcript occurs. The presence of possible biological confounders highlights that comparisons between cancer types should focus on commonalities rather than differences. We further note that the size of the resulting ceRNA networks in SPONGEdb can be increased by adjusting the FDR cutoff, which defaults to 0.05 to provide suitable candidates for experimental validation.

### Cancer subtype analysis of BRCA

To further investigate the influence of cancer subtype-related heterogeneity, we performed independent analyses for the major breast cancer subtypes (luminal A, luminal B, HER2, and basal) from TCGA data. We chose breast cancer since its subtypes are clinically well defined, and since this dataset has a sufficiently high sample number to consider subtypes. In Supplementary Section S9, (Supplementary Table S5 and Figures S3 and 4), we show that, despite the reduced heterogeneity, the number of significant interactions is dramatically reduced as a consequence of the lower sample number.

Next, we subsampled luminal A, luminal B, and basal subtype data as well as the entire BRCA cohort to 132 samples (owing to basal, the smallest of the subtype cohorts considered here). This analysis (Figure 5; Supplementary Section S9, Table S6, Figure S5 and 6) shows that the number of overall and significant interactions differs strongly between subtypes. Luminal A, which is the most frequent and least aggressive subtype, shows the largest number of overall and significant interactions compared to the other subtypes as well as compared to a random sample drawn across all subtypes. This shows that subtype-specific analysis can reduce the influence of sample heterogeneity. Only a few significant interactions are reported in the other subtypes, which suggests that a subtype separation alone does not address all sources of heterogeneity. For example, we could recently show that the luminal and basal breast cancer subtypes in TCGA are confounded by tumor-infiltrating leukocytes (54).

### Experimentally validated ceRNAs in a pan-cancer context

**Figure 3.**
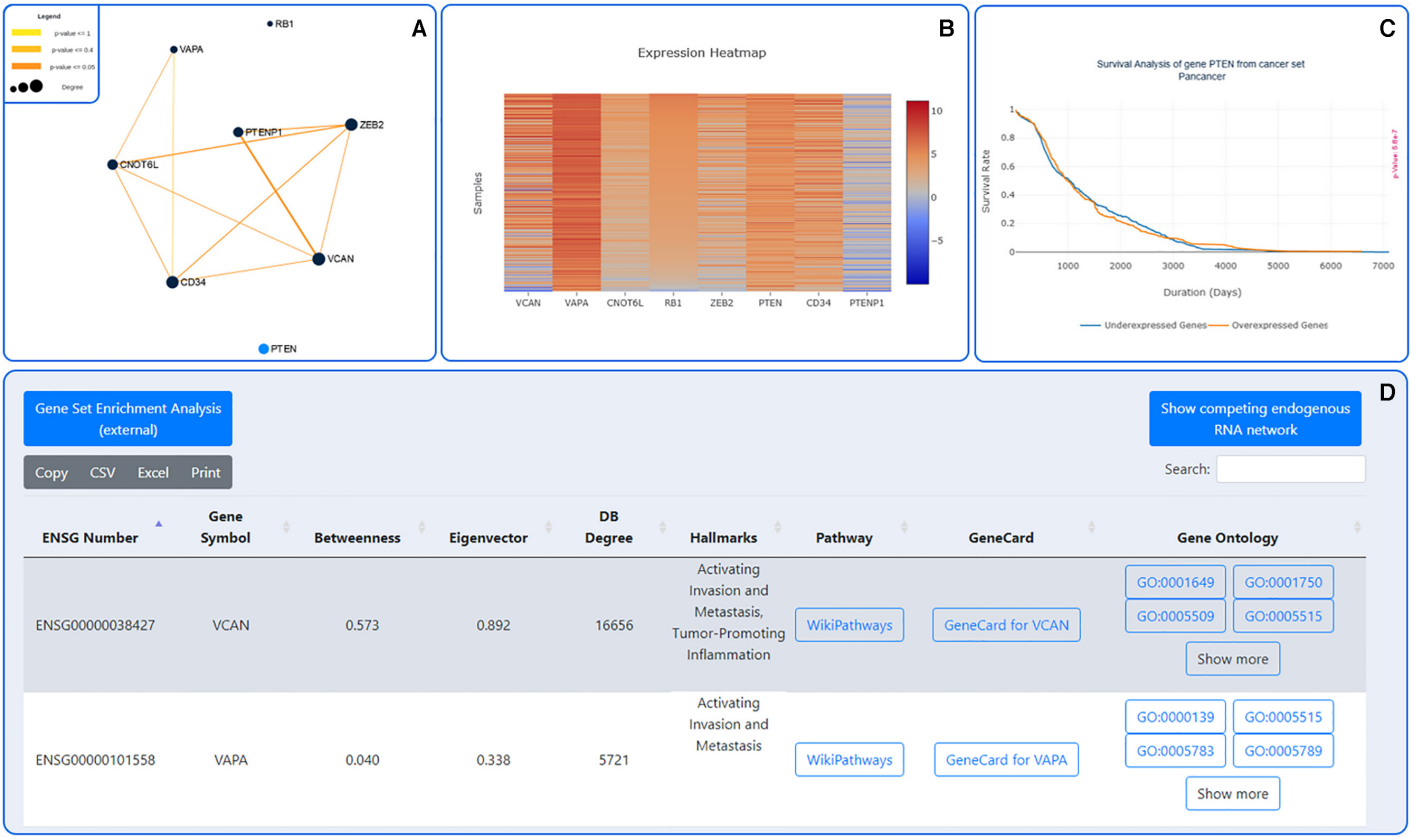
The reproduced figures of PTEN, PTENP1, VCAN, CD34, CNOT6L, VAPA, ZEB2 and RB1. The SPONGE algorithm found most of the described interactions in the Tay *et al.* paper and found additional interactions between the genes, which need to be experimentally validated. Additionally, the website provides following information: (**A**) Network visualizing the interactions between the genes. The edge thickness and color, as well as the node size, corresponds to the *P*-value/node degree. *P*-value ≤ 1: yellow, *P*-value ≤ 0.4: light orange, *P*-value ≤ 0.05: dark orange. (**B**) Expression heatmap. (**C**) Survival Analysis of PTEN. *P*-value = 5.8*e*^−7^. (**D**) External links of the shown genes for one-click further investigation to important web resources. We included WikiPathways, GeneCards, Quick GO and g:Profiler.

SPONGE can also be used to investigate previously reported or putative interactions of experimentally validated ceRNAs. For example, the pseudogene PTENP1 has been reported to regulate levels of the tumor suppressor gene PTEN by competing for shared miRNAs. Low amounts of PTEN are associated with a higher risk of developing cancer. Furthermore, Tay *et al.* reported a dense ceRNA network involving PTEN and PTENP1 together with VCAN, CNOT6L, CD34, VAPA, ZEB2 and RB1. To test if the reported ceRNA network can be reproduced in SPONGEdb, we selected the above query genes in the pan-cancer dataset. The resulting network (Figure 3 A) corroborates previously reported interactions by Tay *et al.* and highlights further putative ceRNA interactions between the selected genes, which need to be experimentally validated.

**Figure 4.**
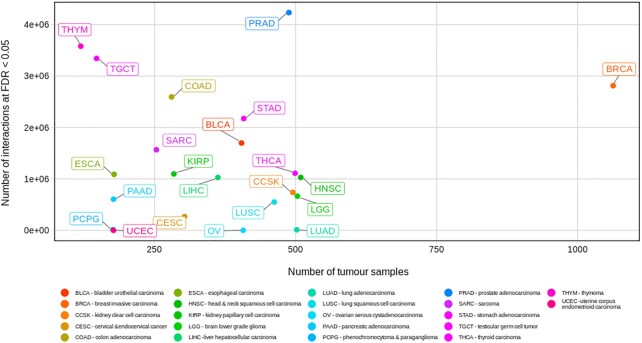
Pan-cancer analysis of the number of significant ceRNA interactions at FDR < 0.05 compared to the number of tumor samples. This figure was created with the SPONGEdb R package, see Supplementary Section S6 for details.

**Figure 5.**
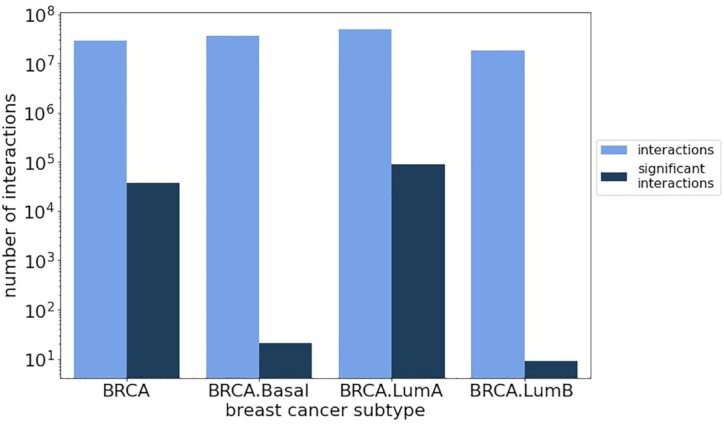
Comparison of results for BRCA and its subtypes luminal A, luminal B and basal after subsampling to 132 samples.

The web interface offers additional insights into the selected network, including a heatmap of expression values (Figure 3B), survival data shown as a Kaplan–Meier plot (Figure 3C), key network centrality measures including degree, betweenness and eigenvector centrality, as well as functional annotation of cancer hallmarks and links to external information about associated pathways and gene ontology terms, including gene set enrichment analysis (Figure 3D). All information shown in the web interface can conveniently be exported in tabular format or as an image.

### Experimentally validated ceRNAs show better sponging capacity than random ceRNAs

**Figure 6.**
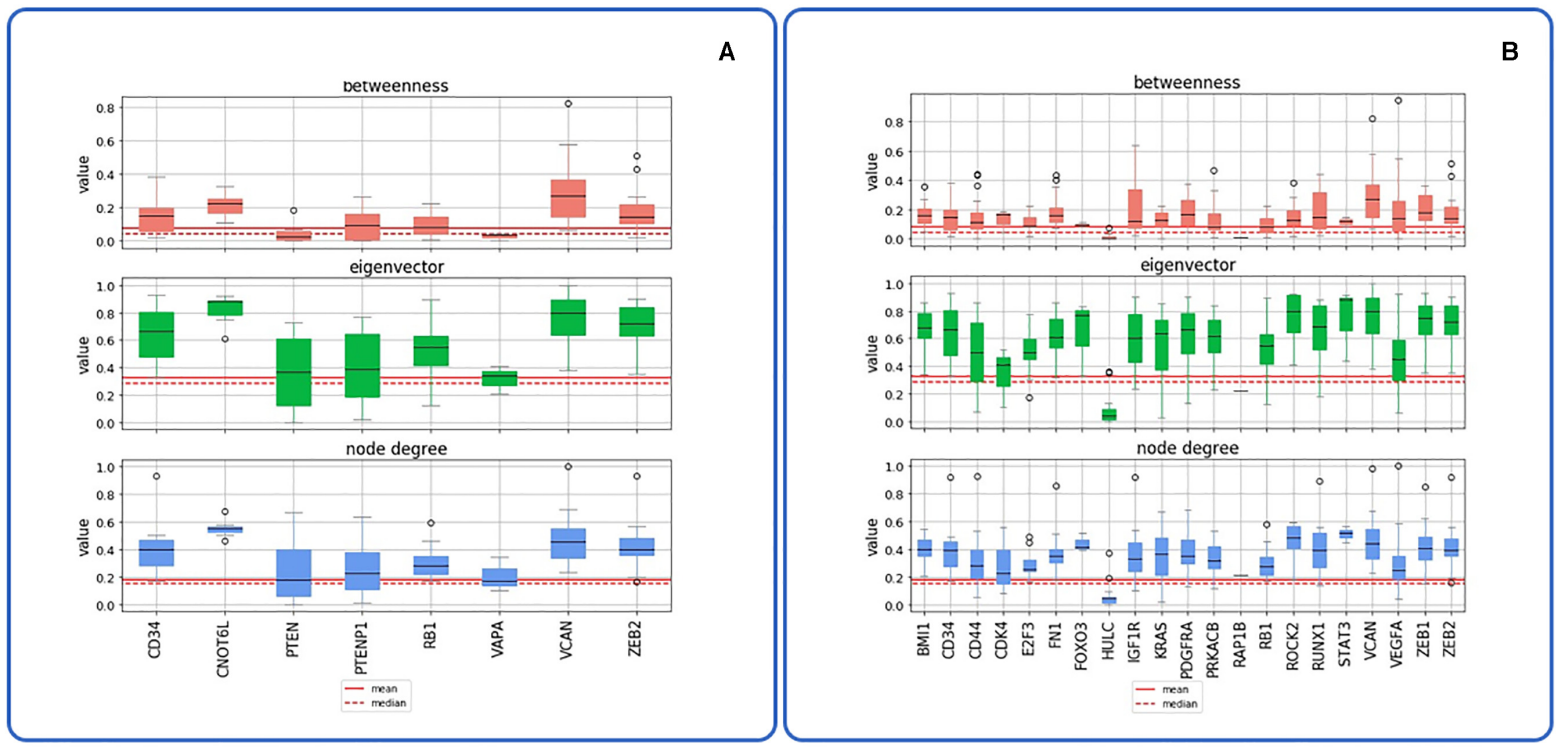
Different network centrality measures summarized across cancer types for experimentally validated ceRNAs from (**A**) Tay *et al.* (33) and (**B**) miRSponge (19). This plot was produced using the SPONGEdb python package (see Supplementary Section S12). Note that the mean and median were computed across all cancer types.

Next, we investigated if experimentally validated ceRNAs outperform random sets of ceRNAs in terms of network centrality measures. For this analysis, we selected the eight ceRNAs reported by Tay *et al.* as well as another 21 experimentally validated ceRNAs reported by miRSponge and compared their centrality measures to an average of 1000 times randomly chosen gene set of equal size as well as to the median and mean of all genes available inside the network. Our results (Supplementary Section S10 and Figures S7–10) show that the betweenness, node degree and eigenvector centralities are considerably higher for these ceRNAs. We also investigated if these ceRNAs are only relevant for specific cancer types by systematically comparing their centrality measures. Our results (Supplementary Section S11, Figures S11 and 12) show that many experimentally validated ceRNAs are relevant in a larger number of cancer types. Figure 6 shows that all investigated ceRNAs but VAPA (Tay *et al.*) and HULC, RAP1B (miRSponge) have centrality measures larger than the mean of all ceRNAs reported in SPONGEdb. This corroborates that network centrality measures reported by SPONGEdb capture meaningful ceRNA biology and that predicted ceRNAs are promising candidates for experimental validation.

## CONCLUSION AND OUTLOOK

To date, TCGA is the most comprehensive resource of molecular profiling data in cancer research (27). The availability of paired gene and miRNA expression data offers a unique chance to comprehensively study ceRNA regulation in a network context. Here, we made SPONGE-inferred ceRNA networks for 22 cancer types available as an easy-to-access resource. To facilitate broad utility, SPONGEdb implements an API that allows biomedical researchers to query the results programmatically to embed our results in their own analyses and tools. Moreover, we offer an interactive web interface to browse, filter and visualize the results. In conclusion, SPONGEdb is an important resource for studying the role of ceRNAs in various cancer types and for prioritizing ceRNA candidates for experimental validation. For the future, we plan to extend SPONGEdb with more sophisticated network analysis features, e.g. for the detection of disease-relevant subnetworks via network enrichment (55). Although the paired gene and miRNA expression data are currently scarce, we envision that such data will become more prevalent, allowing us to extend SPONGEdb beyond the current application in cancer. Moreover, we expect that co-profiling of gene and miRNA expression in single cells will allow us to infer cell-type-specific ceRNA networks to further broaden the scope of SPONGEdb.

## DATA AVAILABILITY

SPONGE website:

http://sponge.biomedical-big-data.de

API:

http://sponge-api.biomedical-big-data.de

R-package:

https://github.com/biomedbigdata/SPONGE-web-R

Python-package:

https://github.com/biomedbigdata/spongeWebPy

Static-file-server:

http://sponge-files.biomedical-big-data.de

## Supplementary Material

zcaa042_Supplemental_File
