# Supplementary material for: SPONGEdb: a pan-cancer resource for competing endogenous RNA interactions
Source: NAR Cancer. 2021 Jan 6;3(1):zcaa042. doi: 10.1093/narcan/zcaa042 (PMC8210024; doi:10.1093/narcan/zcaa042)
Supplement: zcaa042_Supplemental_File [file zcaa042_supplemental_file.pdf]

Supplemental Materials for SPONGEdb: A pan-cancer resource for competing endogenous RNA interactions

Markus Hoffmann [1,†], Elisabeth Pachl [1,†], Michael Hartung [1,†], Veronika Stiegler [1,†],
Jan Baumbach [1], Marcel H. Schulz [2], Markus List [1, *]

November 12, 2020

[1]Chair of Experimental Bioinformatics, Technical University Munich, Munich, Germany
[2]Institute for Cardiovascular Regeneration, Goethe University, Frankfurt am Main, Germany

[†]contributed equally

This PDF file includes:

Architecture of the database
Architecture of the API
General use of the R and python package
Structure of the data at the static-file-server
Details about the webpage
Example code for R and python packages
Comparison to related databases
Analysis of Australian cohort ovarian serous cystadenocarcinoma
Analysis of subtypes of breast invasive carcinoma
Analysis of ceRNA candidates with experimental evidence
Analysis for Individual Cancer Types on Experimental Validated ceRNAs by Tay et al and miRSponge
Python Code for Experimentally Validated ceRNAs of miRSponge and Tay et al.

[*]To whom correspondence should be addressed. Email: markus.list@wzw.tum.de

Table of Contents

1 Architecture of the Database 3

2 Architecture of the API in Detail 3

3 General use of the R and Python Package 4

4 Structure of the Csv Files at the Static-file-server 5

5 Website Details 6

6 Example Code for R and Python Packages 6
 6.1 R Example Code 6
 6.2 Python Example Code 6

7 Comparison to Related Databases 9

8 Analysis of an Australian Cohort for Ovarian Serous Cystadenocarcinoma 12

9 Analysis of Cancer Subtypes of Breast Invasive Carcinoma 12

10 Analysis of ceRNA candidates with experimental evidence 14

11 Analysis for Individual Cancer Types on Experimental Validated ceRNAs by Tay et al and miRSponge 18

12 Python Code for Experimentally Validated ceRNAs of miRSponge and Tay et al. 22

1 Architecture of the Database

The database uses the innodb version 8.0.16 on a MySQL Community Server-GPL license and is based on a Linux server. It contains information from TCGA (cancer information and pan-cancer analysis), ENCODE (additional gene information) and miRbase (additional miRNA information). The database is normalized (third normal form) to prohibit redundancies and inconsistencies. The main table is the dataset table. From one data set there can be several runs with different parameters, which are specified in the run table. In Fig. 1 the general architecture of the database is shown.

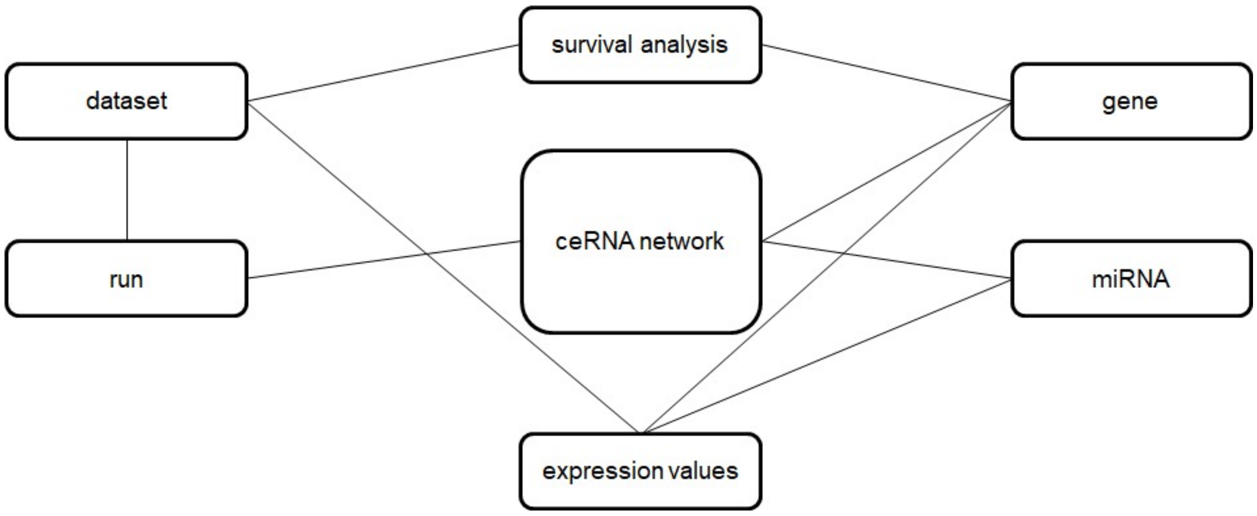

Figure 1: The database architecture - The main table is the data set table in which a cancer is specified. For one cancer type more than one run with different SPONGE parameters can be defined resulting in different ceRNA networks. The gene and miRNA table are filled with all genes and miRNAs found in the ceRNA networks and are filled with additional information about those.

Fig. 2 shows the database in detail. The **dataset** table is the main table of the database. It contains information about the cancer types used in the analysis. The **run** table defines the parameters of the specified actual run in the SPONGE tool. For one dataset there can be various runs, which can differ in the parameters and in the targeted databases, this information is contained in the **target_databases table**. Different parameters can lead to different ceRNA interaction networks as a result of the SPONGE tool. In the **interactions_genegene** table the ceRNA networks are saved. Each gene-gene interaction has a certain P-value, mscor and correlation. The single ceRNA interactions can be selected through those characteristics. The **network_analysis** table describes different characteristics of the ceRNA interaction network like normalized betweenness or degree of each node. Furthermore, the ceRNA interactions can be specified with the miRNAs contributing in this relationship. This data is stored in **interacting_mirnas**. The additional miRNA information is stored in the **mirna** table. The **genes** table contains additional information about the genes used in the ceRNA networks. Input data for SPONGE are paired gene and miRNA expression values. These are stored in the **expression_data_gene** and **expression_data_mirna** table. The **survival_rate** table contains the survival rates of each gene of different cancer types, it is supported by the **suvival_pvalue** table, which retrieves pValues from log rank tests based on raw survival analysis data. The **patient_information** table contains the informations about the patients used in the survival analysis. The **occurence_mirna** table is a table to speed up the access to data for the website. It counts the occurrence of a mirna inside a run. In the **gene_count** table the overall amount of interactions of one gene has in one run is stored. In addition the number of significant interactions with a pValue lower than 0.05 is stored. Internal search indices fasten up the database and therefore reduce waiting time for answering requests, especially with the size of the interactions_genegene table. The tables **gene_ontology**, **wikipathways** and **hallmarks** contain the respective data to form links or information to external resources.

2 Architecture of the API in Detail

The API was built with Flask Version 1.1.1 on Python 3.7. The Representational State Transfer Application Programming Interface (REST-API) uses HTTP to GET, PUT, POST and DELETE data. GET is used to retrieve a resource. PUT changes the state or updates a resource, which can be an object, file or block. POST can create a resource and DELETE removes it. A restful system consists of a client who requests resources and a server who has the resources. Different architectural constraints were considered, like having a 'Uniform Interface' (UI). That means the resources are uniquely identifiable through a single URL, and only by using the underlying methods of the network protocol, such as GET. Moreover, all client-server operations are stateless, and any state management that is required takes place on the client. For the server we used a FLASK application, as it is flexible, minimalistic without losing power, routing the URLs is uncomplicated and easily extensible, which is a great advantage in terms of maintenance, if more endpoints and thus functionality are added to new web resource.
The static-file-server contains data of all cancer types produced by SPONGE and a combined file with added significant interacting miRNAs. The static-file-server can be accessed via the web page.

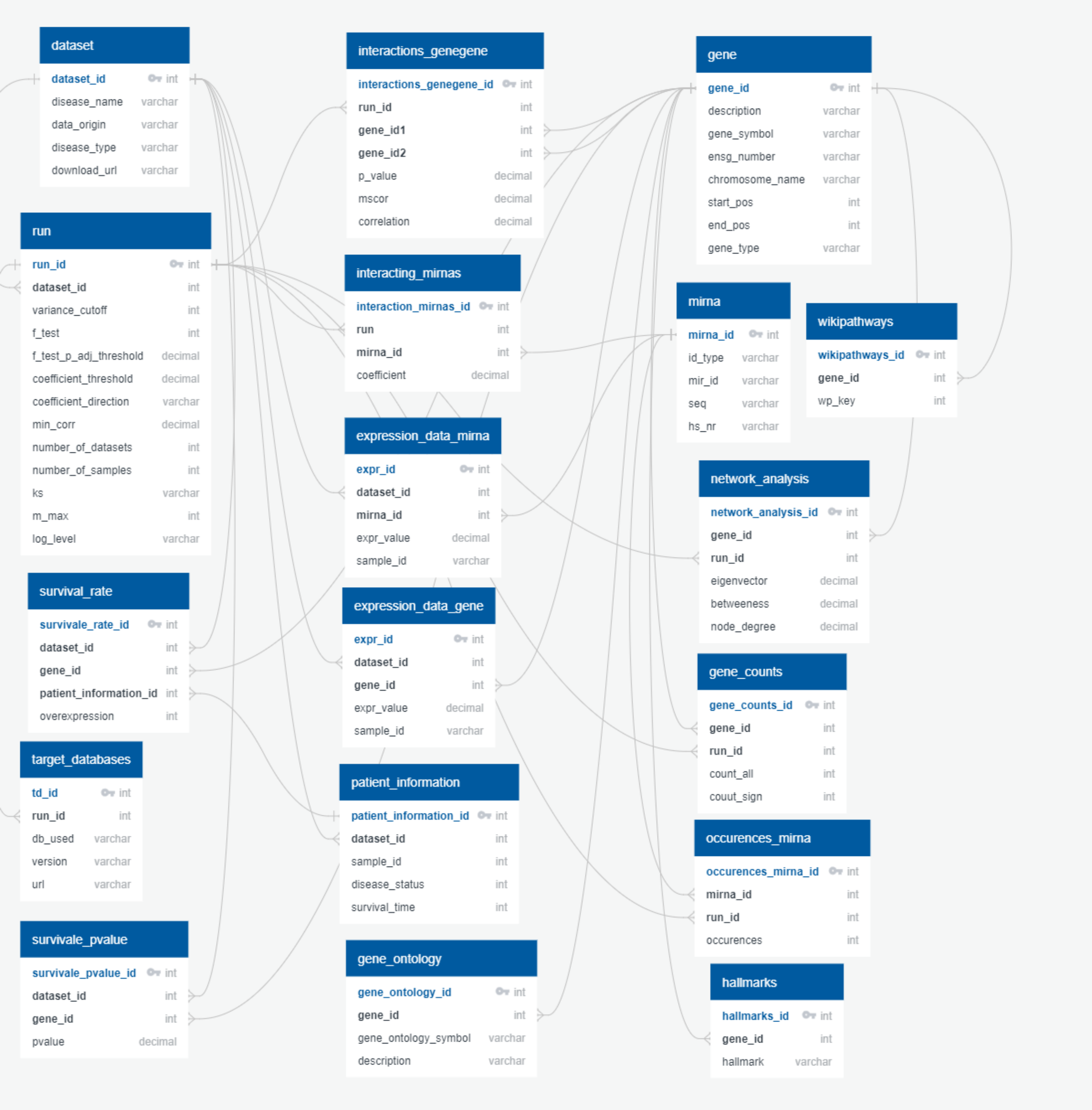

Figure 2: Overview over the database tables

The API is splitted in five head points, which have different endpoints inside to access the data of the database. For the detailed architecture please look at the documentation at: https://exbio.wzw.tum.de/sponge-api/ui/.

3 General use of the R and Python Package

R package: install.packages("spongeWebR")
Python package: pip install spongeWebPy

Both packages have the same functionality and function names inclusive parameter settings. The main difference is the way to use list parameters like ensg_number, gene_symbol, mimat_number, hs_number or sample_ID. The python package uses lists ["x"] and the R packages uses vectors c("x"). In the following we will describe just the python syntax, but R is equivalent.

To start further analysis with SPONGE data, it is important to get an overview about the available disease_types. This can be retrieved with:

get_datasetInformation().

To retrieve all used parameters of the SPONGE method to re-create published results for the cancer type of interest, use the following function:

get(disease_name = "cancertype").

Another way to get an overview of the results is to search for a specific gene and get an idea in which ceRNA interaction network the gene of interest contributes most to:

get_geneCount(gene_symbol = ["genesymbol"]).

To find a sub network of nodes of interest use the functions: Get all ceRNA interactions by given identifications (ensg_number, gene_symbol or gene_type), specific cancer type/dataset or different filter possibilities according different statistical values (e.g. FDR adjusted p-value). Retrieve all possible ceRNAs for gene, identified by ensg_number and threshold for pValue and mscor:

get_all_ceRNAInteractions(ensg_number=[”ENSG1”,”ENSG1”],pValue=0.05, pValueDirection=”<”, mscor=0.006, mscorDirection=”¡”, limit=15).

Get all ceRNAs in a disease of interest (search not for a specific ceRNA, but search for all ceRNAs satisfying filter functions):

get_ceRNA(disease_name = ”cancertype”,gene_type = ”type”, minBetweenness = 0.8)

Get all interactions between the given identifiers (ensg_number or gene_symbol):

get_specific_ceRNAInteractions(disease_name = ”cancertype”,ensg_number = [”ENSG1”,”ENSG2”,”ENSG3”]).

Find sponged miRNAs (the reason for a edge between two ceRNAs) with:

get_sponged_miRNA(disease_name=”kidney”, gene_symbol = [”ENSG1”, ”ENSG2”])

or find a miRNA induced ceRNA interaction. Retrieve all possible ceRNA interactions where miRNA(s) of interest contribute to:

get_specific_miRNAInteraction(disease_name = ”cancertype”, mimat_number = [”MIMAT1”, ”MIMAT2”],limit = 15).

The database also contains information about the raw expression values and survival analysis data, which can be used to for Kaplan-Meyer-Plots (KMPs) for example. These information can be addressed with package functions. To retrieve expression data use:

Retrieve gene expression values for specific genes by ensg_numbers:

get_geneExprValues(disease_name = ”cancertype”, ensg_number = [”ENSG1”,”ENSG2”])

Retrieve gene expression values for specific miRNAs by mimat_numbers:

get_mirnaExprValues(disease_name = ”cancertype”, mimat_number = [”MIMAT1”, ”MIMAT2”])

To get survival analysis data use the function:

get_survAna_rates(disease_name=”cancertype”, ensg_number=[”ENSG1”, ”ENSG2”], sample_ID = [”sample_ID”])

It returns a data_frame with gene and patient/sample information and the ”group information” encoded by column ”overexpressed”. Information about expression value of the gene (FALSE = underexpression, gene expression ¡= mean gene expression over all samples, TRUE = overexpression, gene expression >= mean gene expression over all samples). For further patient/sample information:

get_survAna_sampleInformation(disease_name = ”cancertype”, sample_ID = [”sample_ID”])

4 Structure of the Csv Files at the Static-file-server

For each cancer type a zip file is available. Each zip file contains four csv files:

*cancertype*_interacting_miRNAs.csv

This file contains the interacting miRNAs between two genes with the following headers: geneA, geneB, miRNA, total_number_miRNAs, targetscanA, targetscanB, mircodeA, mircodeB, mirtarbaseA, mirtarbaseB, lncbaseA, lncbaseB.

*cancertype*_interactionNetwork.csv

This file contains the gene gene interaction network with the following headers: geneA, geneB, df, cor, pcor, mscor, p.val, p.adj.

*cancertype*_networkAnalysis.csv

This file contains the network analysis with the following headers: gene, degree, eigenvector, betweenness, page_rank.

*cancertype*_networkInteractions_full.csv

This file contains a mix of the interacting_miRNAs.zip and the interactionNetwork.zip with the following headers: GeneA, GeneB, df, cor, pcor, mscor, p.val, p.adj, miRNA. Only the significant miRNAs are mentioned in a comma separated list.

5 Website Details

On top of that, the general design was made with help of the bootstrap framework, which provided basic structures, such as the footer and the header and additionally many other predefined CSS-classes. Additional JS libraries were installed to achieve different functionalities on the website. The tool jQuery offers a multitude of JS functions and is a dependency for various other JS libraries. The library DataTables offers all necessary operations to make big data tables easily manageable, such as filter and search operations. Sigma js package provided the functionalities for the networks. The Sigma js network visualizes gene-gene interactions, where edges and nodes can be searched and coloured and the created network can be downloaded. Furthermore, Force Atlas 2 was implemented to automatically group nodes in the network. On top of that, we used the graphing library Plotly.js to visualize additional information like general database statistics or even more detailed information about the genes such as expression heatmaps and survival analysis.
The website is designed as a single page application (SPA), which means it is composed from one page. This approach has different advantages. Instead of all components of the website, only single components must be reloaded. Moreover, the website is easy to deploy and to version. The website consists of following sites: Tutorial, Home, Browse, Info and Download.

6 Example Code for R and Python Packages

6.1 R Example Code

```
# import libaries
library(spongeWeb)
library(data.table)
library(ggplot2)
library(ggrepel)

# get number of interactions per dataset with FDR < 0.05
plot_info <- as.data.table(
                  get_overallCounts()[,c("count_interactions_sign","disease_name")])

# get all run_informations for each datasets, which include the sample_size
plot_info[, number_of_samples :=
              unlist(sapply(disease_name, get_runInformation)["number_of_samples",])]

# remove pancancer from data
plot_info <- plot_info[disease_name != "pancancer"]

# sort data by disease name
plot_info <- plot_info[order(plot_info$disease_name),]

# abbreviations for better labeling
plot_info[, abbr := c("BLCA", "LGG", "BRCA", "CESC", "COAD", "ESCA", "HNSC",
                       "CCSK", "KIRP", "LIHC", "LUAD", "LUSC", "OV", "PAAD",
                       "PCPG", "PRAD", "SARC", "STAD", "TGCT", "THYM", "THCA", "UCEC")]

ggplot(plot_info, aes(x=number_of_samples, y=count_interactions_sign,
                       label=abbr, color=abbr, fill =
                       paste(abbr, disease_name, sep = '\t-\t'))) +
                       geom_point(size = 2.5) +
                       guides(colour=FALSE) +
                       geom_text_repel(size = 6) +
                       scale_fill_discrete('Disease Names',
                       guide = guide_legend(override.aes = list(alpha = 0),
                       title.hjust = .4,
                       title.theme = element_text(size=11,face="bold"),
                       label.theme=element_text(size=10), ncol = 1)) +
                       theme(axis.text=element_text(size=20),
                       axis.title=element_text(size=22,face="bold"),
                       legend.key = element_blank()) +
                       theme_bw() +
                       labs(x = "number of tumour samples",
                       y = "number of interactions at FDR < 0.05")
```

6.2 Python Example Code

```
import networkx as nx, matplotlib.pyplot as plt
from spongeWebPy import *

# get top 10 hub genes (highest degree) from breast invasive carcinoma
topGenes =
```

```
    get_ceRNA(disease_name="breast invasive carcinoma", sorting="degree", limit=10)

# get all significant interactions between top genes
data = get_specific_ceRNAInteractions(disease_name="breast invasive carcinoma",
                                      gene_symbol=topGenes["gene.gene_symbol"],
                                      pValue=0.05, limit=100)

# create edge list for graph
elist = tuples =
    [tuple(x) for x in data[['gene1.gene_symbol', 'gene2.gene_symbol']].values]
G = nx.Graph()
G.add_edges_from(elist)


# draw graph
pos = nx.circular_layout(G)
#nx.draw(G, pos=pos, node_size=500)
#Create the plot
Xv=[pos[k][0] for k in pos]
Yv=[pos[k][1] for k in pos]
Xed=[]
Yed=[]
for edge in G.edges:
    Xed+=[pos[edge[0]][0],pos[edge[1]][0], None]
    Yed+=[pos[edge[0]][1],pos[edge[1]][1], None]

trace1=go.Scatter(x=Xed, y=Yed,
    line=dict(width=0.5, color='black'),
    text=['PPP1R12B', 'ABCA9', 'DLC1', 'TCF4',
          'LTBP2', 'ARHGAP20', 'ADGRA2', 'LAMA2', 'PLEKHH2', 'FAT4'],
    mode='lines')



trace1.text=['PPP1R12B', 'ABCA9', 'DLC1', 'TCF4', 'LTBP2',
             'ARHGAP20', 'ADGRA2', 'LAMA2', 'PLEKHH2', 'FAT4'],


trace2=go.Scatter(
    x=Xv, y=Yv,
     mode="markers+text",
     name="Markers and Text",
     text=["<b>PPP1R12B</b>", "<b>ABCA9</b>", "<b>DLC1</b>",
           "<b>TCF4</b>", "<b>LTBP2</b>", "<b>ARHGAP20</b>",
           "<b>ADGRA2</b>", "<b>LAMA2</b>", "<b>PLEKHH2</b>",
           "<b>FAT4</b>"],
     textposition="bottom center",
     textfont=dict(
        family="sans serif",
        size=14,
        color="black"
    ),


    marker=dict(
        showscale=True,
        # colorscale options
        #'Greys' | 'YlGnBu' | 'Greens' | 'YlOrRd' | 'Bluered' | 'RdBu' |
        #'Reds' | 'Blues' | 'Picnic' | 'Rainbow' | 'Portland' | 'Jet' |
        #'Hot' | 'Blackbody' | 'Earth' | 'Electric' | 'Viridis' |
        colorscale='Plotly3',
        reversescale=True,
        color=[],
        size=16,
        colorbar=dict(
            thickness=15,
            title='Node Connections',
            xanchor='left',
            titleside='right'
        ),
        line_width=2))
```

```
#color nodes
node_adjacencies = []
node_text = []
for node, adjacencies in enumerate(G.adjacency()):
    node_adjacencies.append(len(adjacencies[1]))
    node_text.append('# of connections: '+str(len(adjacencies[1])))

trace2.marker.color = node_adjacencies
annot=""

data=[trace1, trace2]
fig=go.Figure(data=data,layout=go.Layout(
                #title='<br>Network graph made with Python',
                titlefont_size=16,
                showlegend=False,
                #hovermode='closest',
                margin=dict(b=20,l=5,r=5,t=20),
                annotations=[ dict(
                    showarrow=False,
                    xref="paper", yref="paper",
                    opacity=0.8,
                    x=0.005, y=-0.002 ) ],
    plot_bgcolor='rgba(0,0,0,0)',
                xaxis=dict(showgrid=False, zeroline=False, showticklabels=False),
                yaxis=dict(showgrid=False, zeroline=False, showticklabels=False),

    )

)



fig['layout']['annotations'][0]['text']=annot
fig.show()
```

7 Comparison to Related Databases

Features	SPONGEdb	miRTissue$_{ce}$	LnCeVar	Pan-ceRNADB	miRTarBase
ceRNA interaction database source	DIANA-LnCBase<br>TargetScan<br>miRcode<br>miRTarBase	miRTarBase<br>miRCode<br>LnCACTdb 2.0<br>miRSponge	miRanda<br>mirBase<br>miRTarBase<br>RNAhybrid<br>TargetScan<br>TarBase	TarBase<br>miRTarBase<br>miRecords	HMDD<br>NCBI<br>RefSeq<br>miRBase<br>miRanda<br>TargetScan
ceRNA classes	miRNA-mRNA<br>miRNA-lncRNA<br>miRNA-Pseudogene	miRNA-mRNA<br>miRNA-lncRNA<br>miRNA-Pseudogene	miRNA–mRNA<br>miRNA-lncRNA	miRNA-mRNA	miRNA-mRNA
ceRNA network prediction algorithm	SPONGE	hypergeometric test<br>global test<br>SPONGE	N/A	N/A	N/A
TCGA expression profiles for ceRNA interactions	✓	✓	✓	✓	✗
p value on ceRNA interaction score	✓	✓	✗	✗	✗
Multiple ceRNA analysis	✓	✓	✓	✗	✗
Multiple tissue selection	✗	✓	✓	✗	✗
Pancancer analysis	✓	✗	✗	✗	✗
Hallmarks	✓	✗	✓	✗	✗
Expression data in heatmaps	✓	✗	✗	✗	✗
Survival analysis	✓	✗	✓	✗	✗
Gene Ontology	✓	✓	✓	✗	✗
R / Python packages	✓	✗	✗	✗	✗
API	✓	✗	✗	✗	✗
Interactive network interface	✓	✗	✓	✗	✗
Gene enrichment	✓	✓	✗	✗	✗
References	N/A	[1]	[2]	[3]	[4]

Table 1: Feature comparison between SPONGEdb and ten related databases (part 1/3 with $SPONGEdb, miRTissue_{ce}, LnCeVar, Pan-ceRNADB, miRTarBase$). Partly adopted from miRTissue$_{ce}$ [1].

Features	SPONGEdb	ceRDB	lnCeDB	miRSponge
ceRNA interaction database source	DIANA-LnCBase<br>TargetScan<br>miRcode<br>miRTarBase	TargetScan	TargetScan<br>StarBase<br>miRcode	TarBase<br>miRTarBase<br>miRanda<br>miRecord
ceRNA classes	miRNA-mRNA<br>miRNA-lncRNA<br>miRNA-Pseudogene	miRNA-mRNA<br>miRNA-lncRNA	miRNA-mRNA<br>miRNA-lncRNA	miRNA-mRNA<br>miRNA-lncRNA<br>miRNA-Pseudogene<br>miRNA-circRNA
ceRNA network prediction algorithm	SPONGE	N/A	hypergeometric test	hypergeometric test
TCGA expression profiles for ceRNA interactions	✓	✗	✗	✗
p value on ceRNA interaction score	✓	✗	✗	✗
Multiple ceRNA analysis	✓	✗	✗	✗
Multiple tissue selection	✗	✗	✗	✗
Pancancer analysis	✓	✗	✗	✗
Hallmarks	✓	✗	✗	✗
Expression data in heatmaps	✓	✗	✓	✗
Survival analysis	✓	✗	✗	✗
Gene Ontology	✓	✗	✗	✓
R / Python packages	✓	✗	✗	✗
API	✓	✗	✗	✗
Interactive network interface	✓	✗	✗	✗
Gene enrichment	✓	✗	✗	✓
References	N/A	[5]	[6]	[7]

Table 2: Feature comparison between SPONGEdb and ten related databases (part 2/3 with $SPONGEdb, ceRDB, lnCeDB, miRSponge$). Partly adopted from miRTissue$_{ce}$ [1].

Features	SPONGEdb	LncACTdb	miRcode	starBase v2.0
ceRNA interaction database source	DIANA-LnCBase<br>TargetScan<br>miRcode<br>miRTarBase	miRTarBase<br>miRanda<br>TarBase	TargetScan	miRBase<br>refSeq<br>Ensembl database<br>TargetScan<br>Pictar2, miRanda<br>PITA, RNA22
ceRNA classes	miRNA-mRNA<br>miRNA-lncRNA<br>miRNA-Pseudogene	miRNA-mRNA<br>miRNA-lncRNA<br>miRNA-Pseudogene<br>miRNA-circRNA	miRNA-mRNA<br>miRNA-lncRNA<br>miRNA-Pseudogene	miRNA-mRNA<br>miRNA-lncRNA<br>miRNA-Pseudogene<br>miRNA-circRNA
ceRNA network prediction algorithm	SPONGE	hypergeometric test<br>Pearson correlation	N/A	hypergeometric test
TCGA expression profiles for ceRNA interactions	✓	✓	✗	✓
p value on ceRNA interaction score	✓	✗	✗	✗
Multiple ceRNA analysis	✓	✗	✗	✗
Multiple tissue selection	✗	✗	✗	✗
Pancancer analysis	✓	✓	✗	✗
Hallmarks	✓	✓	✗	✗
Expression data in heatmaps	✓	✗	✗	✗
Survival analysis	✓	✓	✗	✗
Gene Ontology	✓	✓	✗	✗
R / Python packages	✓	✗	✗	✗
API	✓	✗	✗	✗
Interactive network interface	✓	✗	✗	✗
Gene enrichment	✓	✗	✗	✓
References	N/A	[8]	[9]	[10]

Table 3: Feature comparison between SPONGEdb and ten related databases (part 3/3 with $SPONGEdb, LncACTdb, miRcode, starBasev2.0$). Partly adopted from miRTissue$_{ce}$ [1].

8 Analysis of an Australian Cohort for Ovarian Serous Cystadenocarcinoma

	number of samples	total interactions	significant interactions	ratio
TCGA OV*	407	25,023,437	15	0.000001
Australian cohort OV**	82	2,880,023	26	0.00001

Table 4: SPONGE results of an Australian cohort for ovarian serous cystadenocarcinoma compared to the results of the TCGA ovarian cancer cohort.

* https://portal.gdc.cancer.gov/projects/TCGA-OV
** https://dcc.icgc.org/releases/current/Projects/OV-AU

9 Analysis of Cancer Subtypes of Breast Invasive Carcinoma

	BRCA	BRCA.Basal	BRCA.Her2	BRCA.LumA	BRCA.LumB
number of samples	1063	182	80	556	206
total interactions	32,420,434	37,320,655	14,400,514	50,312,893	18,387,833
significant interactions	2,813,051	14	0	95,453	10
ratio	0.0867678	0.0000004	0.0000000	0.0018972	0.0000054
average adj. p-value	0.42	0.47	0.44	0.46	0.45
count common BRCA interactions	-	9,334,075	3,549,913	16,370,216	5,983,780
BRCA average adj. p-value in intersection with BRCA	-	0.38	0.35	0.39	0.36
subtype average adj. p-value in intersection with BRCA	-	0.46	0.45	0.44	0.45

Table 5: SPONGE analysis of subtypes of breast invasive carcinoma

	BRCA	BRCA.Basal	BRCA.LumA	BRCA.LumB
number of samples	132	132	132	132
total interactions	29,607,087	37,247,616	50,262,435	18,518,933
significant interactions	37,742	21	90,543	9
ratio	0.00127476	0.00000056	0.00180140	0.00000049
average adj. p-value	0.41	0.47	0.46	0.45
count common BRCA interactions	-	8,437,176	13,372,139	5,560,593
BRCA average adj. p-value in intersection with BRCA	-	0.40	0.40	0.39
subtype average adj. p-value in intersection with BRCA	-	0.46	0.42	0.45

Table 6: SPONGE analysis of subtypes of breast invasive carcinoma, randomly sub-sampled to 132 samples, excluding Her2

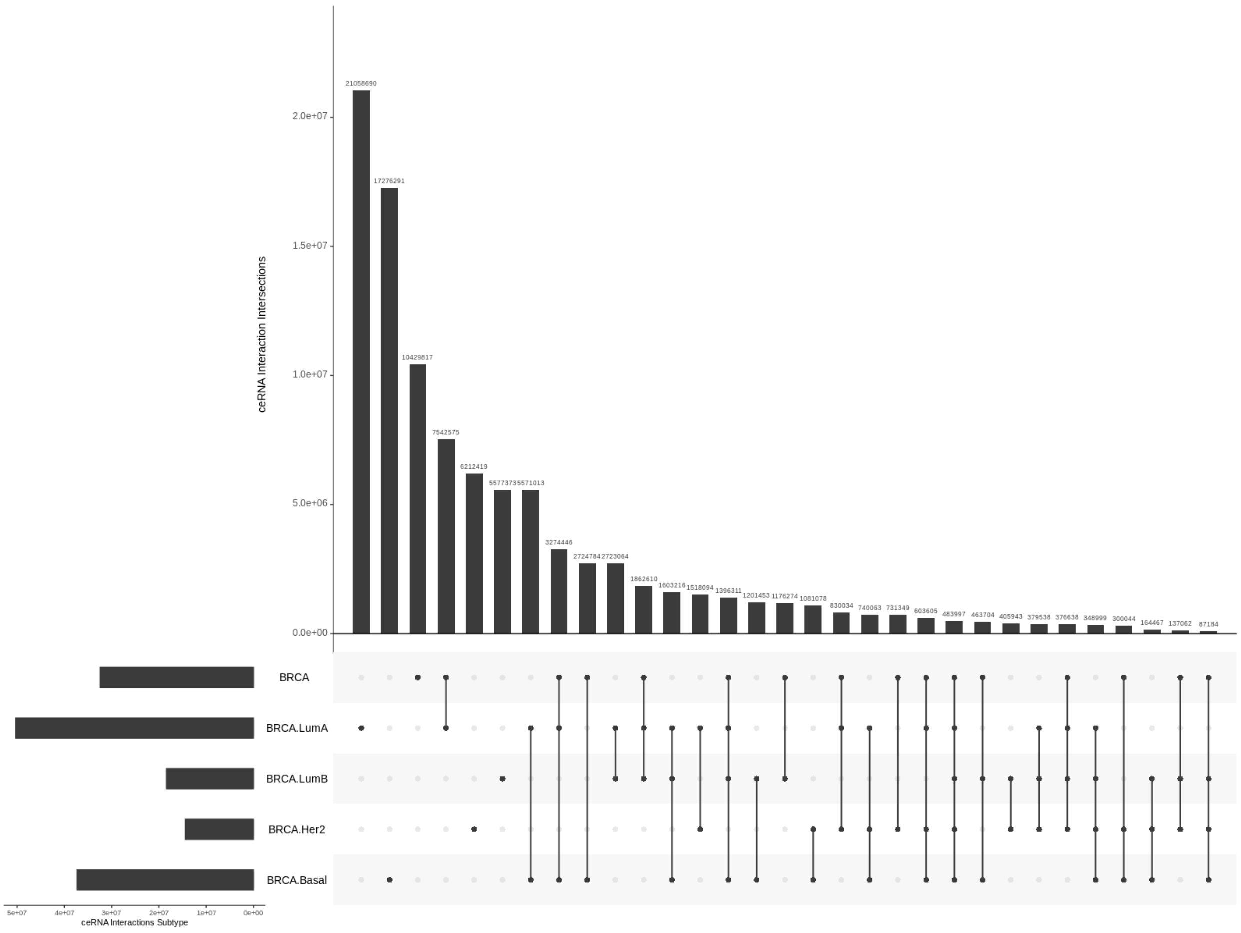

Figure 3: Upset plot showing shared interactions of breast cancer and its subtypes (using all available samples).

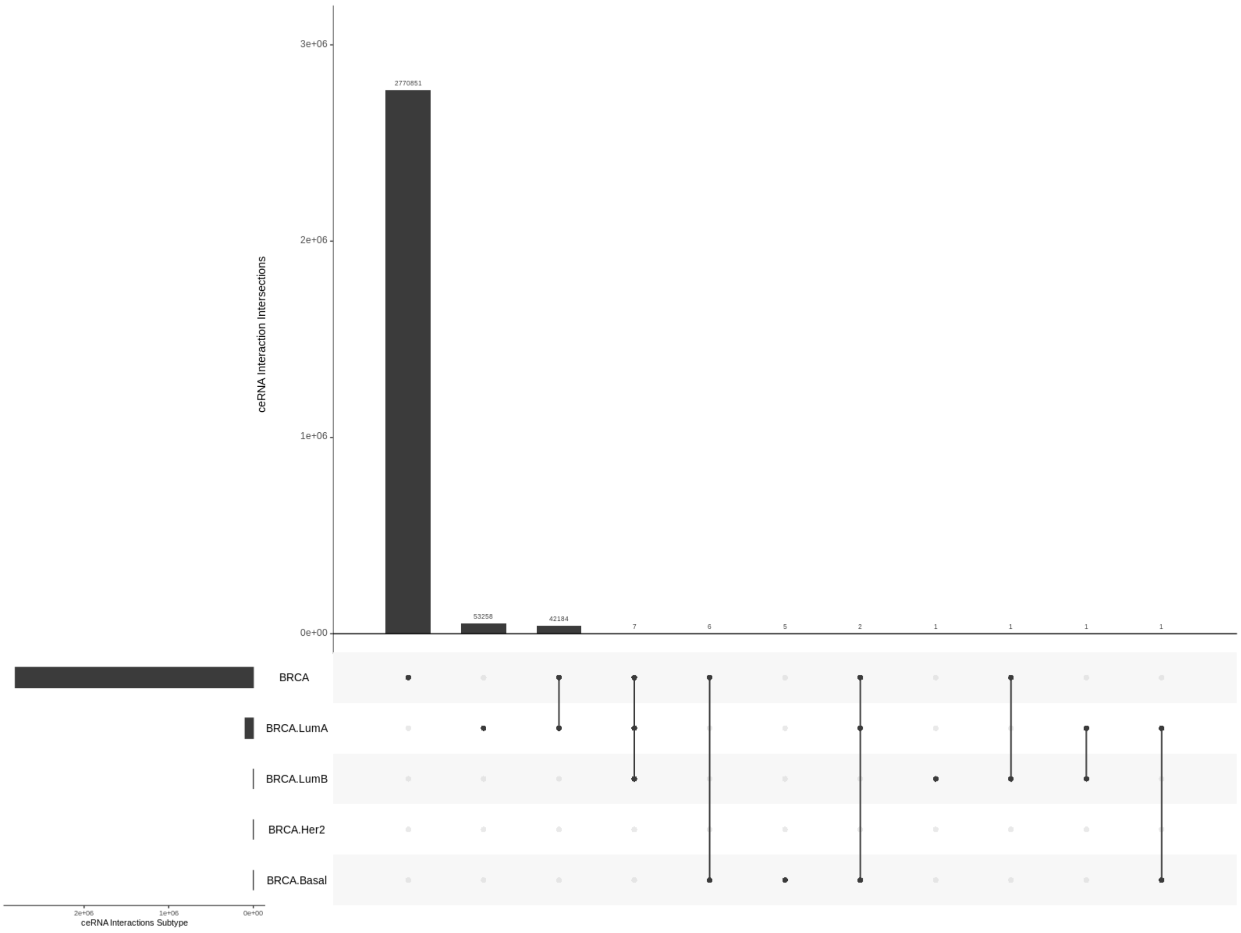

Figure 4: Upset plot showing shared interactions of breast cancer and its subtypes with FDR < 0.05 (using all available samples).

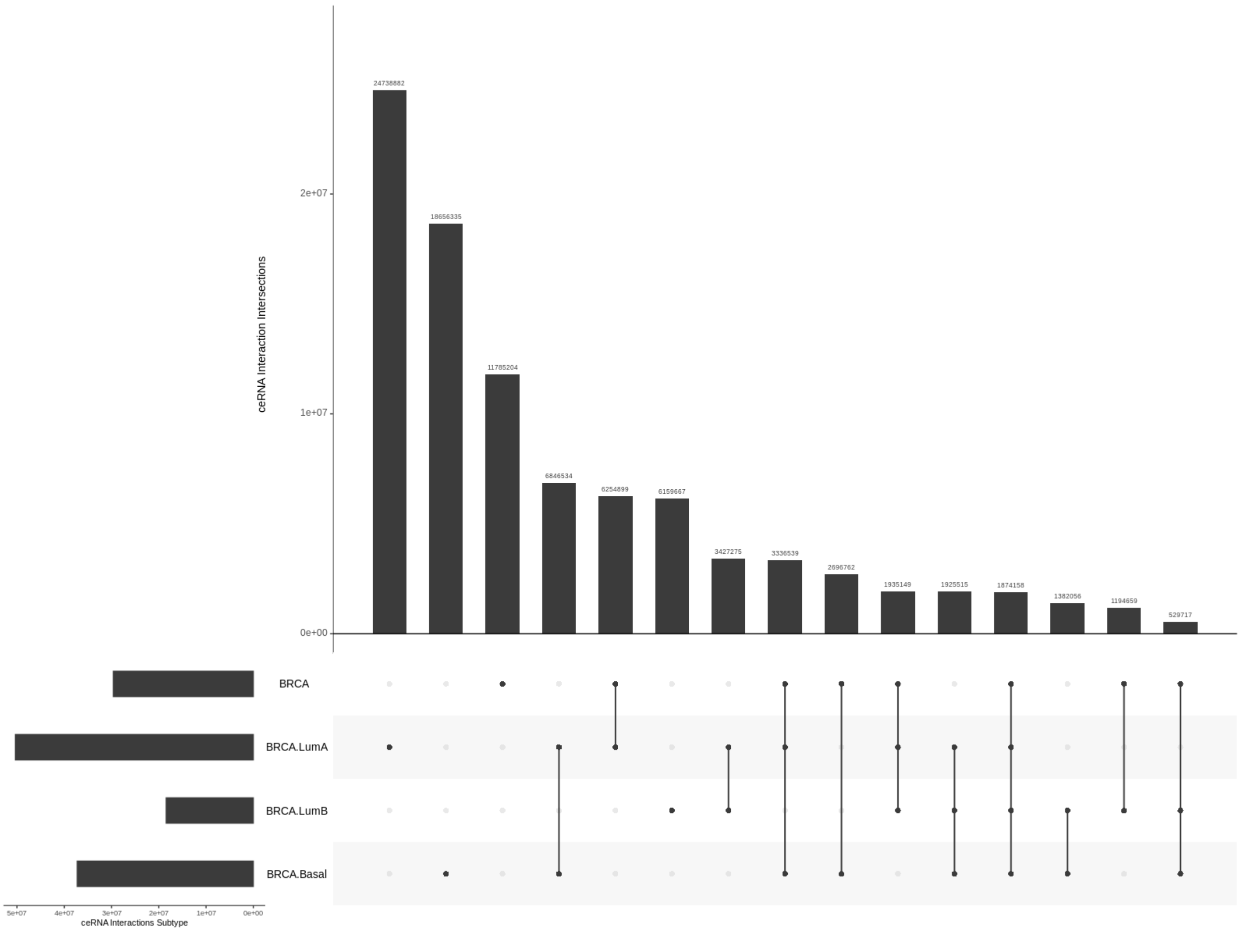

Figure 5: Upset plot showing shared interactions of breast cancer and its subtypes (randomly sub-sampled to 132 samples).

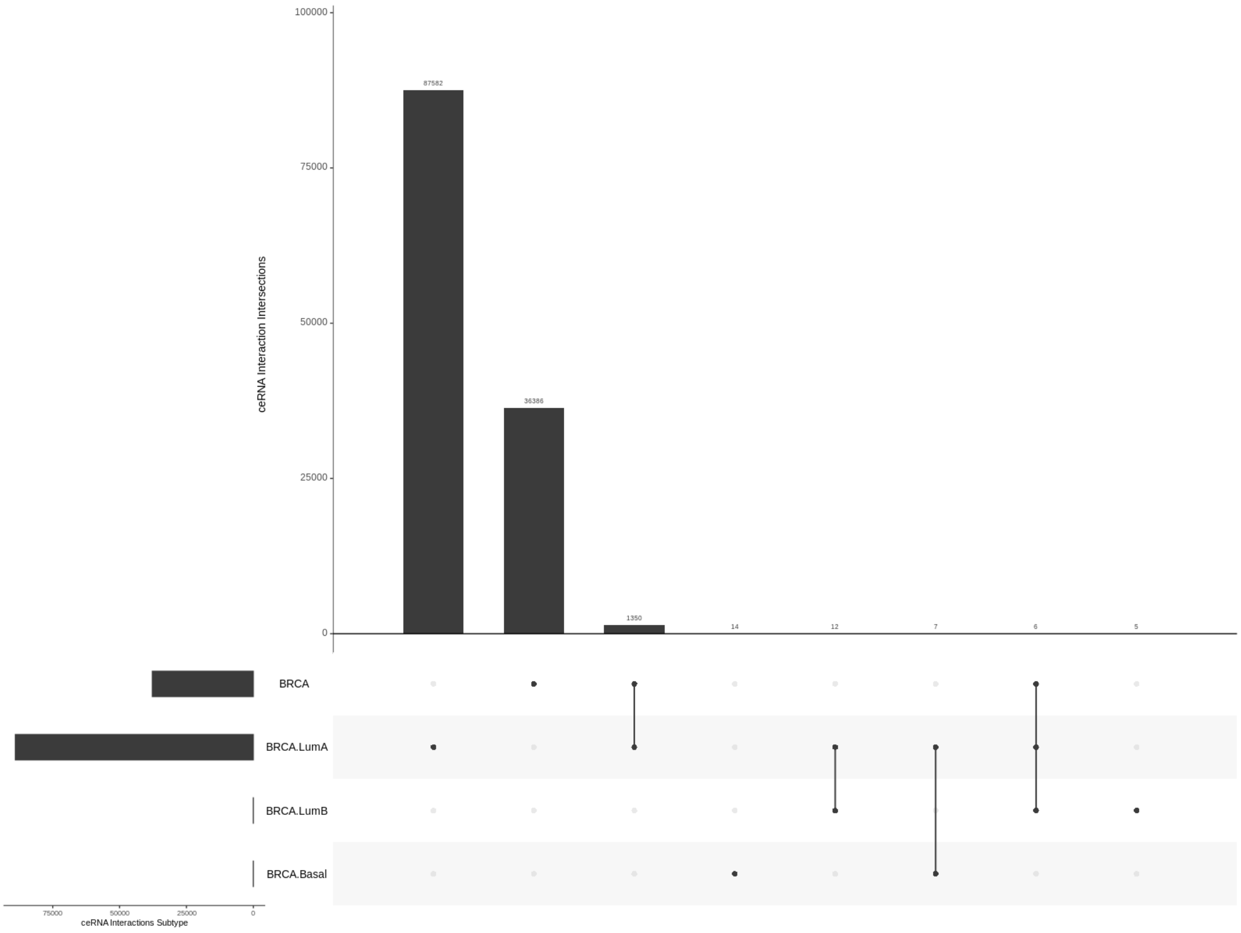

Figure 6: Upset plot showing shared interactions of breast cancer and its subtypes with FDR < 0.05 (randomly sub-sampled to 132 samples).

10 Analysis of ceRNA candidates with experimental evidence

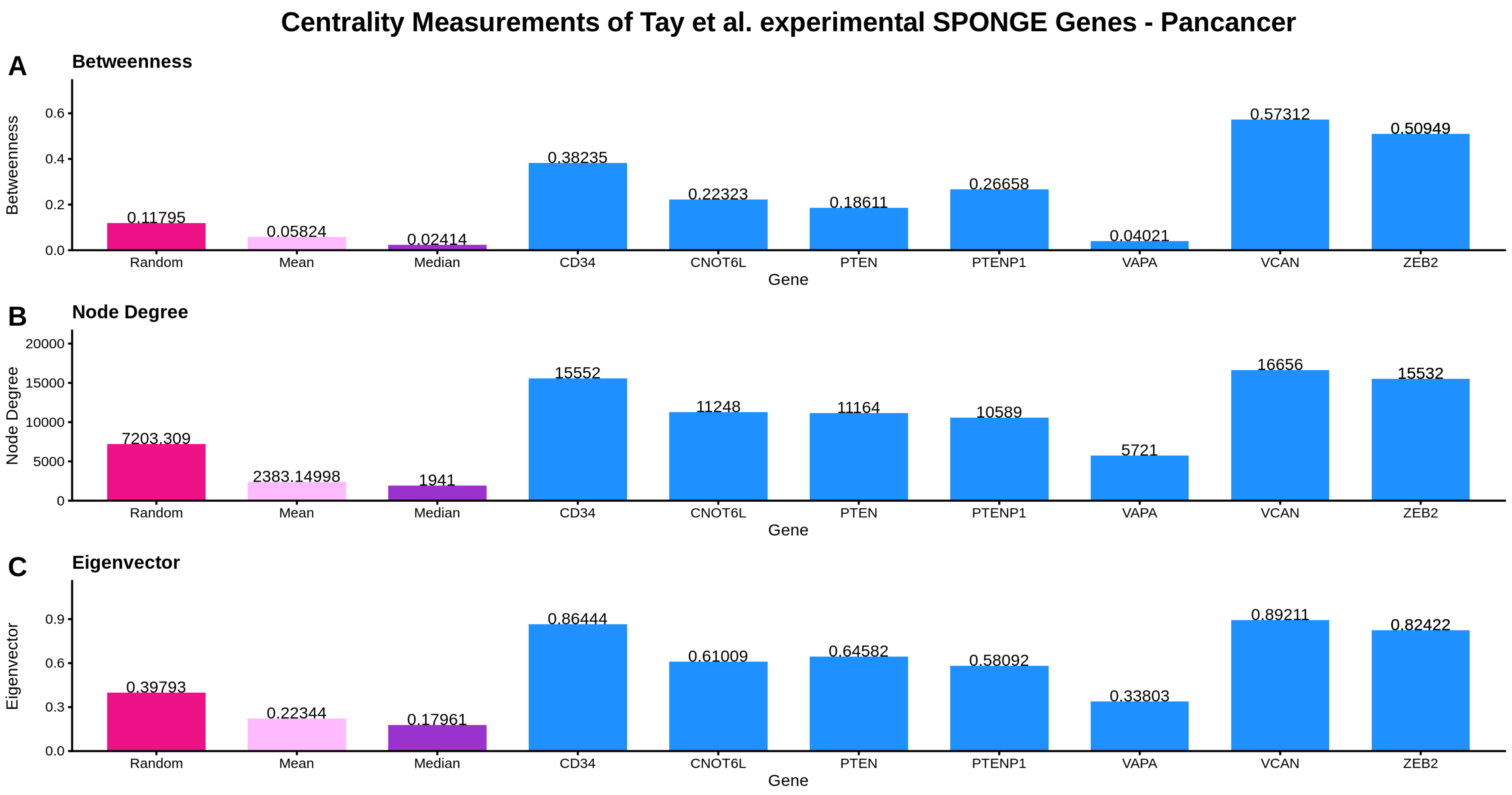

Figure 7: Centrality measures of the ceRNA candidates reported in Tay et al. [11] in the pan-cancer analysis compared to a random selection of 1000 ceRNAs as well as the mean and median values of all available genes.

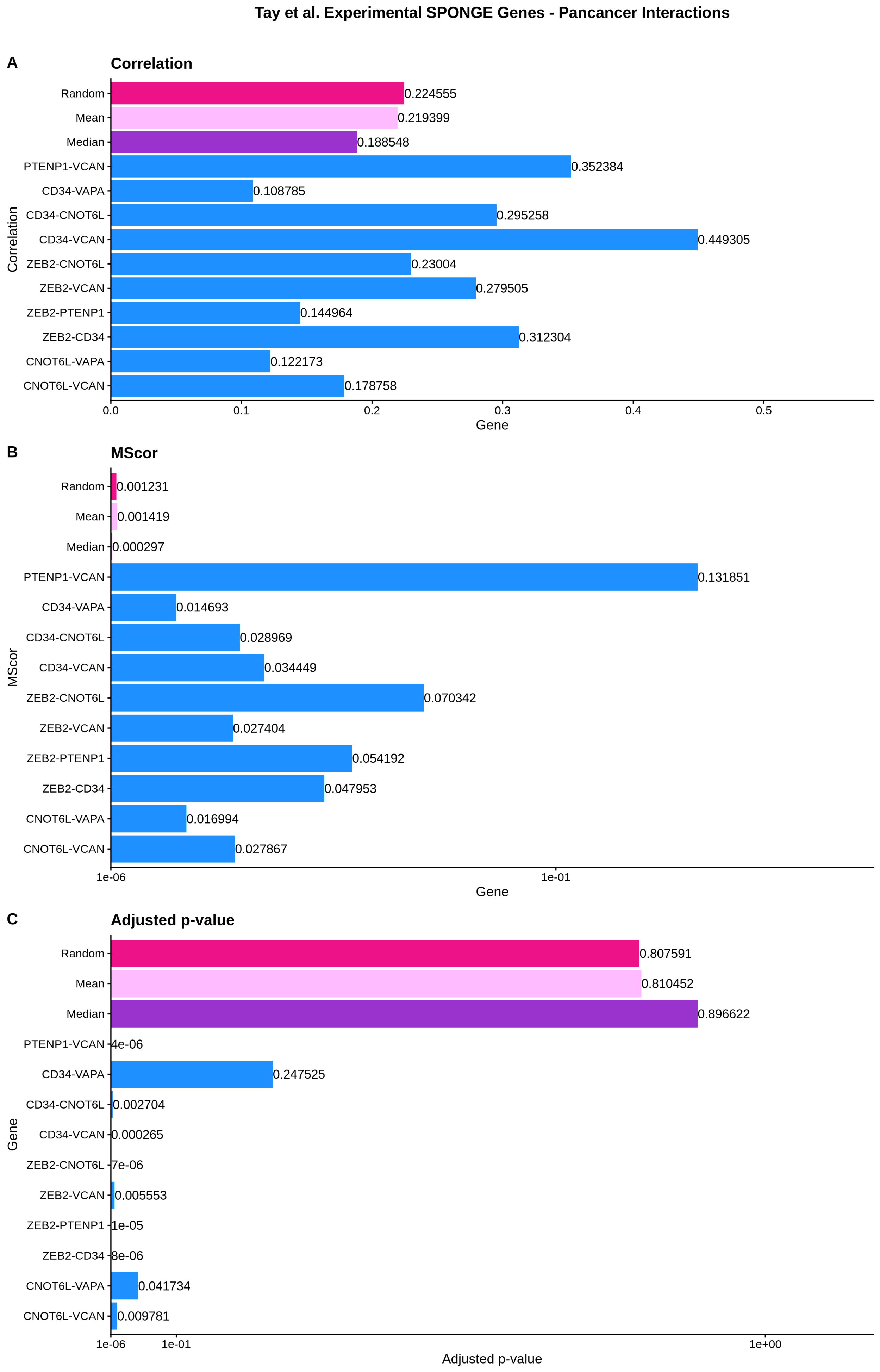

Figure 8: Correlation, MScor and adjusted p-value of ceRNA candidates in Tay et al. [11] in the pan-cancer analysis compared to a random selection of 1000 ceRNA interactions as well as the mean and median values of all available interactions.

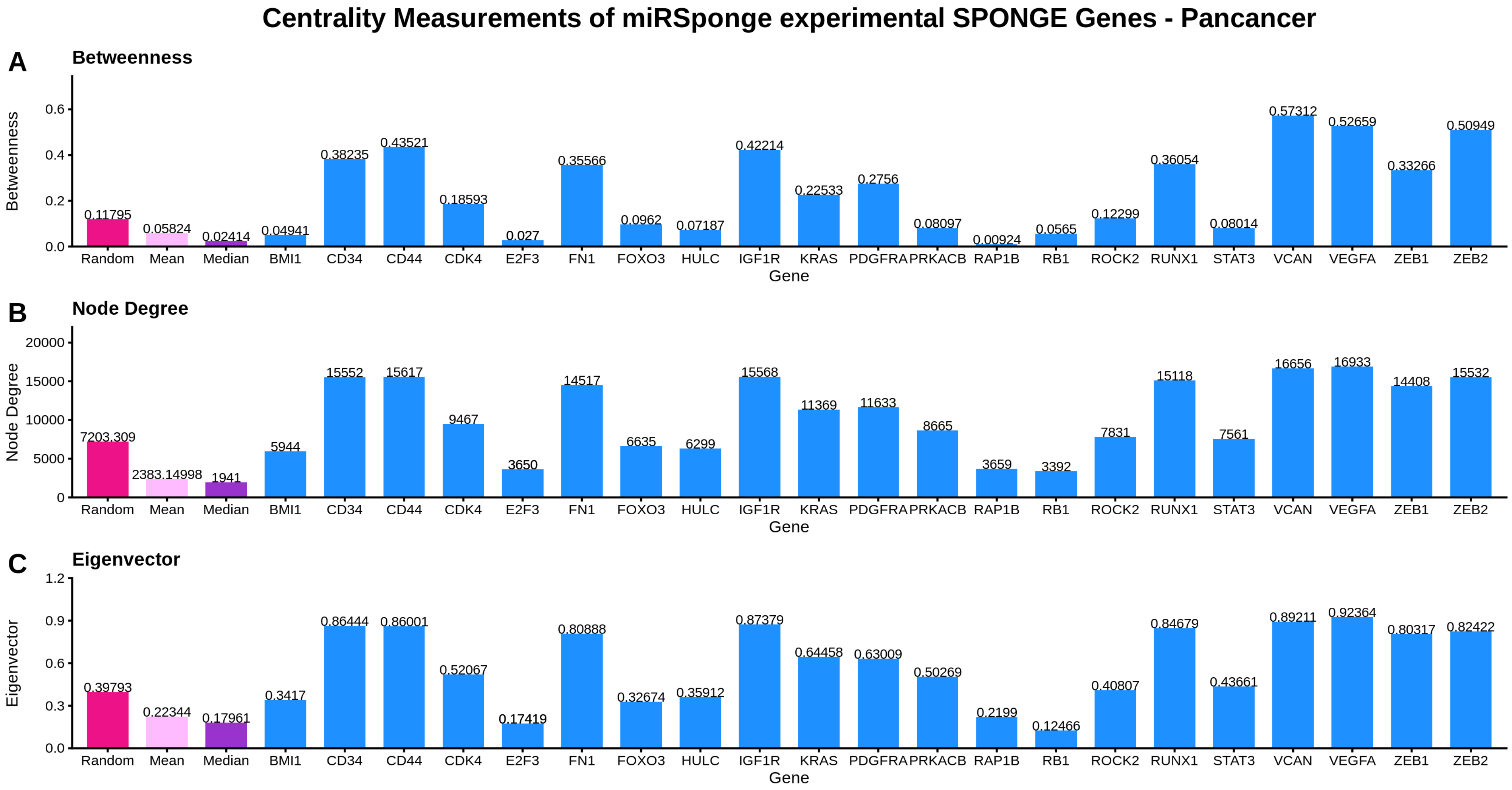

Figure 9: Centrality measures of ceRNA candidates with experimental evidence according to miRSponge [7] in the pan-cancer analysis compared to a random selection of 1000 ceRNAs as well as the mean and median values of all available genes.

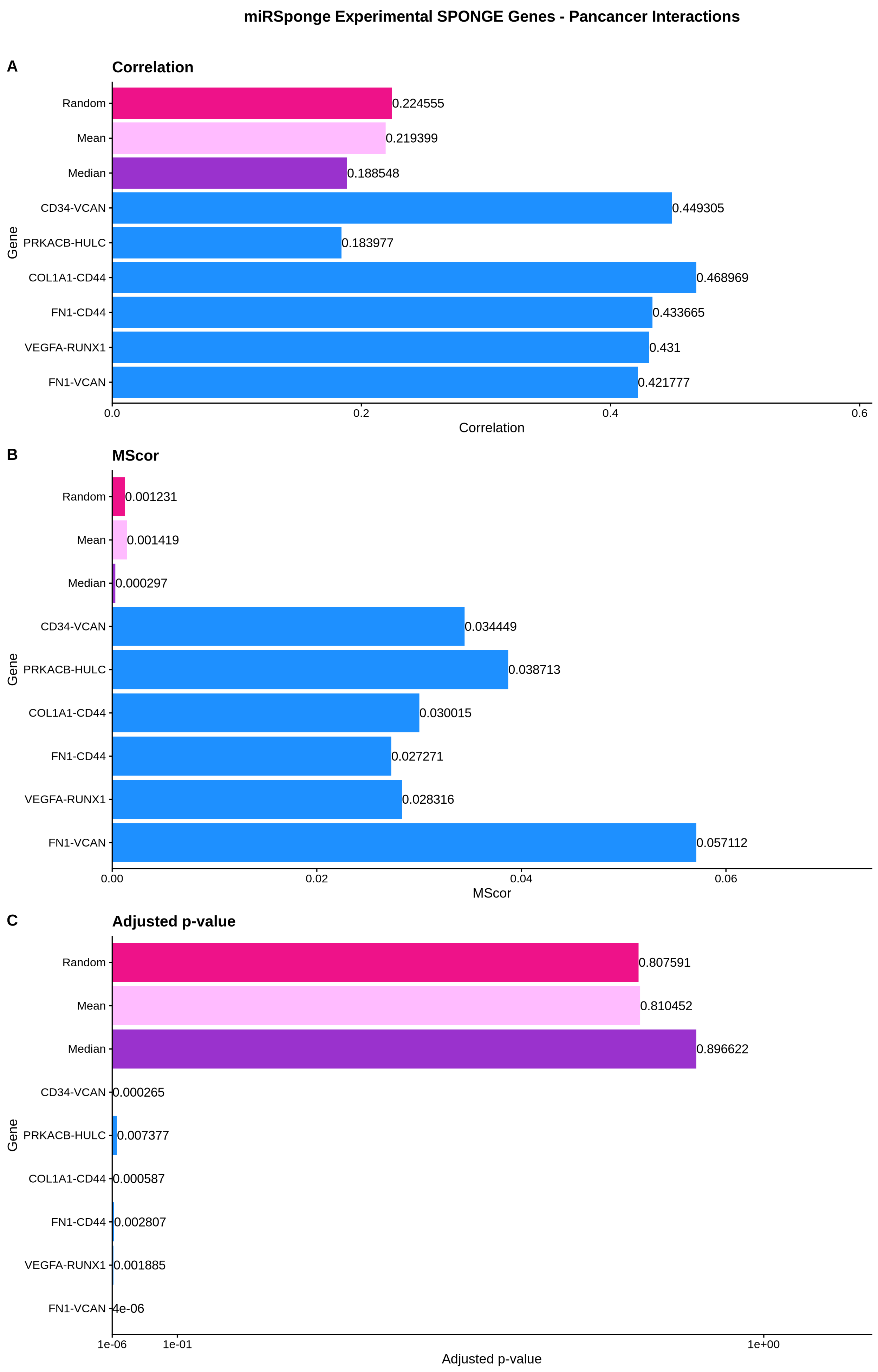

Figure 10: Correlation, MScor and adjusted p-value of ceRNA candidates with experimental evidence according to miRSponge [7] in the pan-cancer analysis compared to a random selection of 1000 ceRNA interactions as well as the mean and median values of all available interactions.

11 Analysis for Individual Cancer Types on Experimental Validated ceRNAs by Tay et al and miRSponge

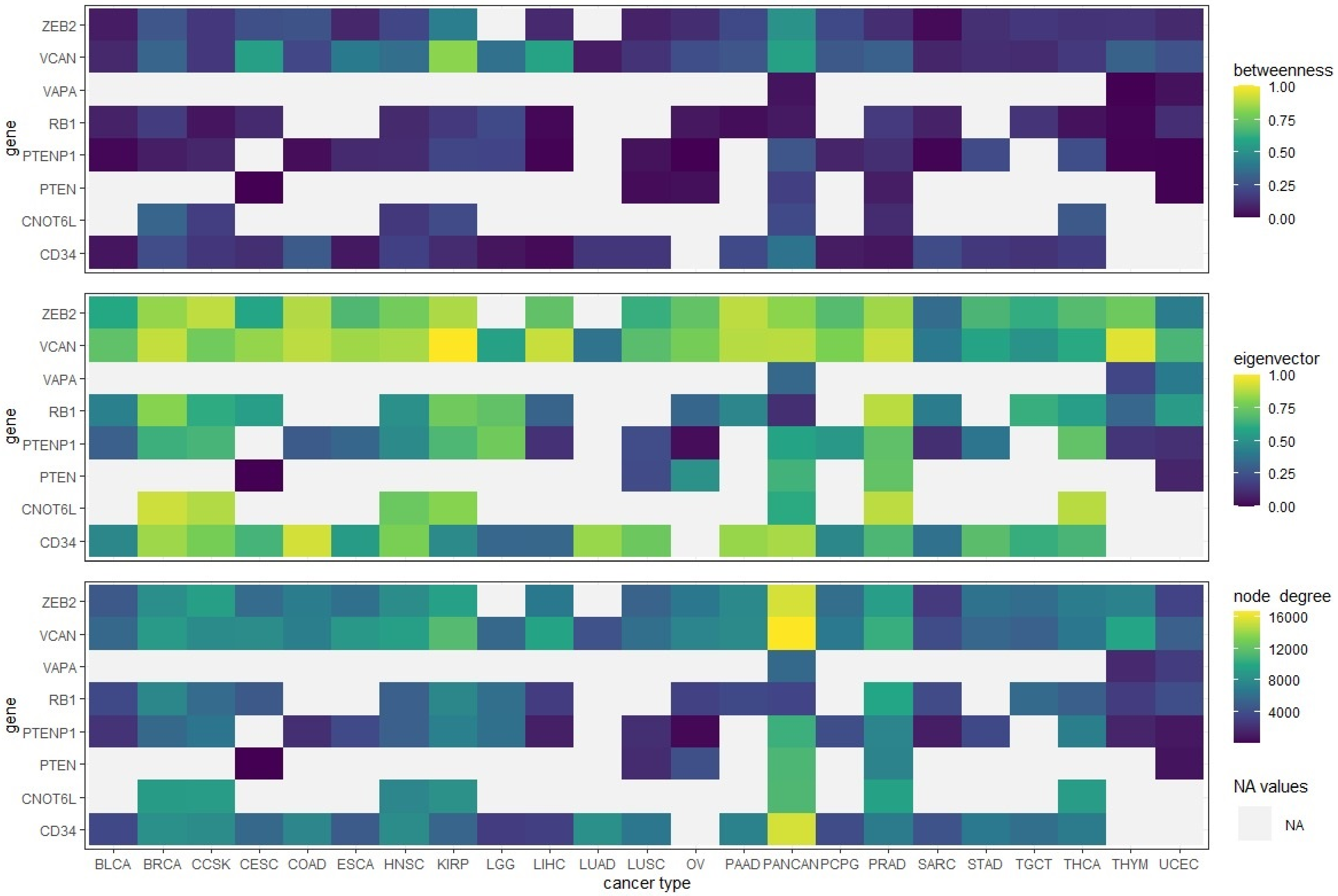

Figure 11: Heatmap of centrality measures of ceRNA candidates reported in Tay et al. [11] across cancer types

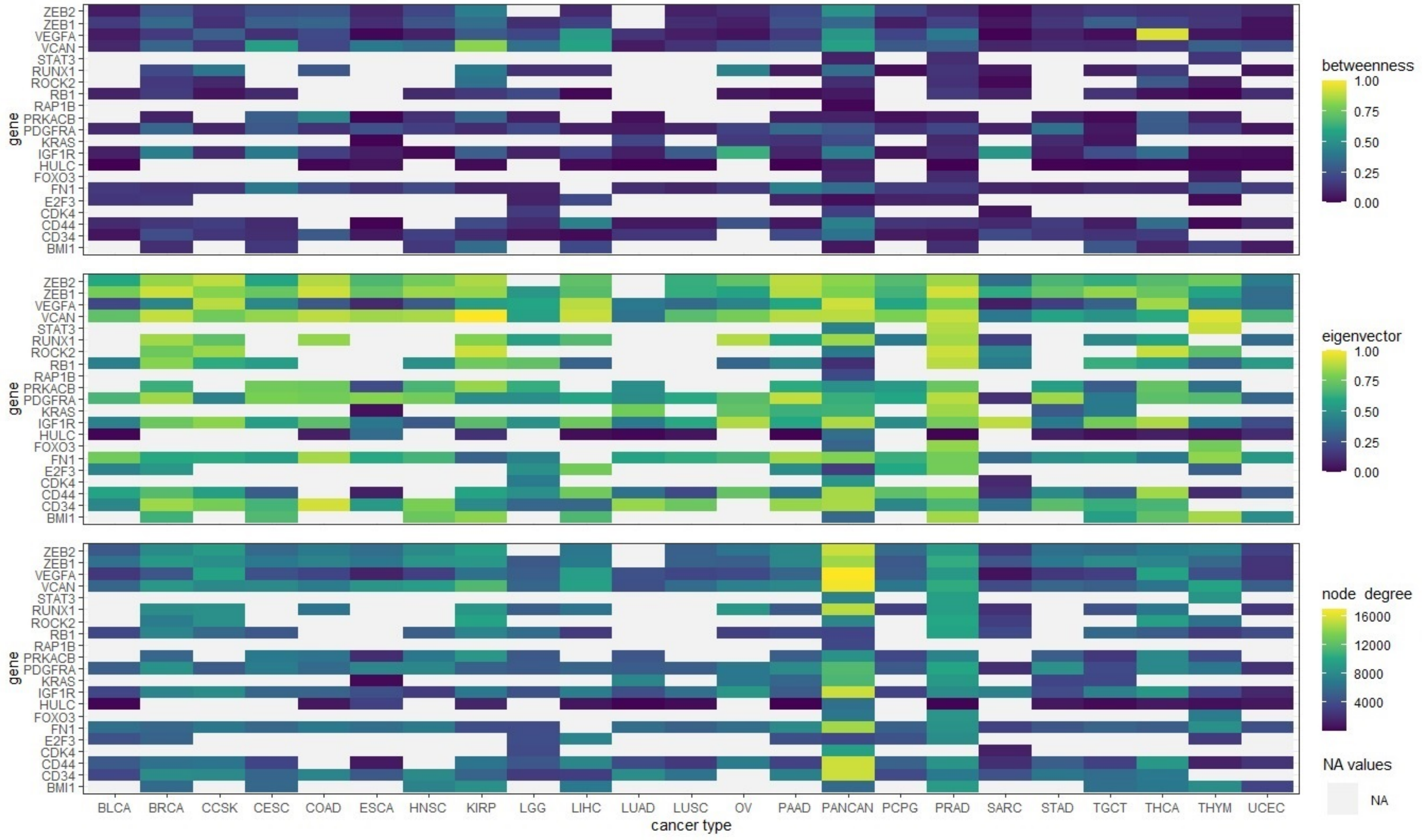

Figure 12: Heatmap of centrality measures of ceRNA candidates according to miRSponge [7] across cancer types.

Example code how to produce the Tay et al. heatmap:

```
library(ggplot2)
library(spongeWeb)
library(plyr)
library(tidyr)
library(egg)

# get network measures for all genes for all cancer types
gene_symbols <- c("PTEN", "PTENP1", "VCAN", "CD34", "CNOT6L", "VAPA", "ZEB2", "RB1")
cancer_types <- sort(get_datasetInformation()["disease_name"][1:23,])

values <- data.frame("disease_name"=rep(cancer_types, each=length(gene_symbols)),
"gene_symbol"=rep(gene_symbols, times=length(cancer_types)),
"betweenness"=c(NA), "eigenvector"=c(NA), "node_degree"=c(NA))

for (i in cancer_types){
  for (j in gene_symbols){

  tryCatch(
    {
     tmp <- get_ceRNA(disease_name=i, gene_symbol=j)[c(1,2,5)]
    },
    error = function(e){
      tmp <- c(NA, NA, NA)
    }
  )

  values[which((values$disease_name == i) & (values$gene_symbol == j)), 3:5] <- tmp
  }
}

# add abbrevation for cancer for better labeling
abbr <- c( "BLCA", "LGG", "BRCA", "CESC", "COAD", "ESCA", "HNSC",
           "CCSK", "KIRP", "LIHC", "LUAD", "LUSC", "OV", "PANCAN",
           "PAAD","PCPG", "PRAD", "SARC", "STAD", "TGCT", "THYM", "THCA",
           "UCEC" )
values$abbr <- mapvalues(values$disease_name, from = cancer_types, to = abbr)

# from wide to long format
long <- values %>% gather(measure, value, -c(disease_name, gene_symbol, abbr))

# create datasets with wanted limits
myLimits <- list(
  list("betweenness", 0, 1),
  list("eigenvector", 0, 1),
  list("node_degree", min(values$node_degree), max(values$node_degree))
)


plotHeat <- function(type, MIN, MAX) {
  library(ggplot2)
  p <- ggplot(subset(long, measure == type),
              aes(abbr, gene_symbol, fill = value, label = "")) +
    geom_tile() +
    geom_text(color = "black", size = 3) +
    scale_fill_continuous(type = "viridis", limits = c(MIN, MAX)) +
    labs(x    = "cancer_type",
         y    = "gene",
         fill = type) +
    theme_bw()
  # Output x-axis only for the last plot
  if (type != myLimits[[length(myLimits)]][[1]]) {
    p <- p + theme(axis.text.x = element_blank(),
                   axis.title.x = element_blank(),
                   axis.line.x = element_blank(),
                   axis.ticks.x = element_blank())
  }
  return(p)
}

res <- lapply(myLimits, function(x) {plotHeat(x[[1]], x[[2]], x[[3]])})
egg::ggarrange(plots = res)
```

12 Python Code for Experimentally Validated ceRNAs of miR-Sponge and Tay et al.

```
import spongeWebPy as sponge
import pandas as pd
import matplotlib.pyplot as plt
import numpy as np

# genes to analyse
# Tay et al.
gene_symbols = ["PTEN", "PTENP1", "VCAN", "CD34",
                "CNOT6L", "VAPA", "ZEB2", "RB1"]

#miRSponge
#gene_symbols <- ["BMI1", "CD34", "CD44", "CDK4",
#                 "E2F3", "FN1", "FOXO3", "HULC",
#                 "IGF1R", "KRAS", "PDGFRA", "PRKACB",
#                 "RAP1B", "RB1", "ROCK2", "RUNX1",
#                 "STAT3", "VCAN", "VEGFA", "ZEB1",
#                 "ZEB2"]

# get all cancer types without ovarian cancer (in alphabetical order)
cancer_types = sponge.get_datasetInformation()["disease_name"][0:23,].sort_values()

# all available information from all cancer type for all genes
network_measures = []
for i, x in enumerate(cancer_types):
    print(str(i) + ", " + x)
    offset = 0
    limit = 1000
    tmp = sponge.get_ceRNA(disease_name=x, limit=limit, offset=offset)
    network_measures.append(tmp)

    while len(tmp) == limit:
        offset = offset + limit
        network_measures.append(tmp)
        tmp = sponge.get_ceRNA(disease_name=x, limit=limit, offset=offset)

    # add last tmp
    network_measures.append(tmp)

network_measures = pd.concat(network_measures)

# normalize node_degree
network_measures["node_degree"] = network_measures["node_degree"]
/max(network_measures["node_degree"])

# use spongeWeb to retrieve network centrality measures
values = [sponge.get_ceRNA(disease_name=x, gene_symbol=gene_symbols, limit=200)
[["betweenness", "eigenvector", "gene.gene_symbol", "node_degree",
"run.dataset.disease_name"]]
for x in cancer_types]

values = pd.concat(values)

#normalize node degree values
values["node_degree"] = values["node_degree"]/max(values["node_degree"])

# add abbrevation for cancer for better labeling
abbr = ["BLCA", "LGG", "BRCA", "CESC", "COAD", "ESCA", "HNSC",
        "CCSK", "KIRP", "LIHC", "LUAD", "LUSC", "OV", "PANCAN",
        "PAAD","PCPG", "PRAD", "SARC", "STAD", "TGCT", "THYM", "THCA",
        "UCEC"]
values["abbr"] =
values['run.dataset.disease_name'].map(dict(zip(cancer_types.values, abbr)))


# plot information gene-wise
fig, (ax1, ax2, ax3) = plt.subplots(3, sharex=True, figsize=(10,10))

# colorblind friendly colors
```

```
CB_color_cycle = ['#ef7c71', '#07b542', '#689bee']

# set up subplots
props = dict(boxes=CB_color_cycle[0], whiskers=CB_color_cycle[0], medians="Black",
caps="Gray")
values.boxplot(column=["betweenness"], by="gene.gene_symbol",
ax=ax1, color=props, patch_artist=True)
ax1.set_xlabel("")
ax1.set_ylabel("value", fontsize=15)
ax1.tick_params(axis="y", which="major", labelsize=13)
ax1.set_title("betweenness", fontsize=17)

#add mean and median for all genes in database
all_mean = np.mean(network_measures["betweenness"])
all_median = np.median(network_measures["betweenness"])
ax1.hlines(y=all_mean, xmin=0, xmax=ax1.get_xlim()[1],
linestyles="solid", color="red", label="mean")
ax1.hlines(y=all_median, xmin=0, xmax=ax1.get_xlim()[1],
linestyles="dashed", color="red", label="median")

props = dict(boxes=CB_color_cycle[1],
whiskers=CB_color_cycle[1], medians="Black", caps="Gray")
values.boxplot(column=["eigenvector"], by="gene.gene_symbol",
ax=ax2, color=props, patch_artist=True)
ax2.set_xlabel("")
ax2.set_ylabel("value", fontsize=15)
ax2.tick_params(axis="y", which="major", labelsize=13)
ax2.set_title("eigenvector", fontsize=17)

#add mean and median for all genes in database
all_mean = np.mean(network_measures["eigenvector"])
all_median = np.median(network_measures["eigenvector"])
ax2.hlines(y=all_mean, xmin=0, xmax=ax2.get_xlim()[1],
linestyles="solid", color="red", label="mean")
ax2.hlines(y=all_median, xmin=0, xmax=ax2.get_xlim()[1],
linestyles="dashed", color="red", label="median")

props = dict(boxes=CB_color_cycle[2],
whiskers=CB_color_cycle[2], medians="Black", caps="Gray")
values.boxplot(column=["node_degree"], by="gene.gene_symbol",
ax=ax3, color=props, patch_artist=True, rot=90)
ax3.set_xlabel("")
ax3.set_ylabel("value", fontsize=15)
ax3.tick_params(axis="x", which="major", labelsize=15)
ax3.tick_params(axis="y", which="major", labelsize=13)
ax3.set_title("node degree", fontsize=17)

#add mean and median for all genes in database
all_mean = np.mean(network_measures["node_degree"])
all_median = np.median(network_measures["node_degree"])
ax3.hlines(y=all_mean, xmin=0, xmax=ax3.get_xlim()[1],
linestyles="solid", color="red", label="mean")
ax3.hlines(y=all_median, xmin=0, xmax=ax3.get_xlim()[1],
linestyles="dashed", color="red", label="median")

# add legend
handles, labels = ax1.get_legend_handles_labels()
fig.legend(handles, labels, loc='lower center')

# show figure
plt.show()
```

References

[1] Antonino Fiannaca, Laura La Paglia, Massimo La Rosa, Riccardo Rizzo, and Alfonso Urso. miRTissue ce: extending miRTissue web service with the analysis of ceRNA-ceRNA interactions. *BMC Bioinformatics*, 21(Suppl 8):199, September 2020.

[2] Peng Wang, Xin Li, Yue Gao, Qiuyan Guo, Shangwei Ning, Yunpeng Zhang, Shipeng Shang, Junwei Wang, Yanxia Wang, Hui Zhi, Ying Fang, Weitao Shen, Guangmei Zhang, Steven Xi Chen, and Xia Li. LnCeVar: a comprehensive database of genomic variations that disturb ceRNA network regulation. *Nucleic Acids Res.*, 48(D1):D111–D117, January 2020.

[3] Juan Xu, Yongsheng Li, Jianping Lu, Tao Pan, Na Ding, Zishan Wang, Tingting Shao, Jinwen Zhang, Lihua Wang, and Xia Li. The mRNA related ceRNA–ceRNA landscape and significance across 20 major cancer types. *Nucleic Acids Res.*, 43(17):8169–8182, August 2015.

[4] Hsi-Yuan Huang, Yang-Chi-Dung Lin, Jing Li, Kai-Yao Huang, Sirjana Shrestha, Hsiao-Chin Hong, Yun Tang, Yi-Gang Chen, Chen-Nan Jin, Yuan Yu, Jia-Tong Xu, Yue-Ming Li, Xiao-Xuan Cai, Zhen-Yu Zhou, Xiao-Hang Chen, Yuan-Yuan Pei, Liang Hu, Jin-Jiang Su, Shi-Dong Cui, Fei Wang, Yue-Yang Xie, Si-Yuan Ding, Meng-Fan Luo, Chih-Hung Chou, Nai-Wen Chang, Wen-Lian, Tzong-Yi Lee, Feng-Xiang Wei, and Hsien-Da Huang. miRTarBase 2020: updates to the experimentally validated microRNA-target interaction database. *Nucleic Acids Res.*, 48(D1):D148–D154, January 2020.

[5] Aaron L Sarver and Subbaya Subramanian. Competing endogenous RNA database. *Bioinformation*, 8(15):731–733, August 2012.

[6] Shaoli Das, Suman Ghosal, Rituparno Sen, and Jayprokas Chakrabarti. lnCeDB: database of human long noncoding RNA acting as competing endogenous RNA. *PLoS One*, 9(6):e98965, June 2014.

[7] Peng Wang, Hui Zhi, Yunpeng Zhang, Yue Liu, Jizhou Zhang, Yue Gao, Maoni Guo, Shangwei Ning, and Xia Li. miRSponge: a manually curated database for experimentally supported miRNA sponges and ceRNAs. *Database*, 2015, September 2015.

[8] Peng Wang, Xin Li, Yue Gao, Qiuyan Guo, Yanxia Wang, Ying Fang, Xueyan Ma, Hui Zhi, Dianshuang Zhou, Weitao Shen, Weisha Liu, Lihua Wang, Yunpeng Zhang, Shangwei Ning, and Xia Li. LncACTdb 2.0: an updated database of experimentally supported ceRNA interactions curated from low- and high-throughput experiments. *Nucleic Acids Res.*, 47(D1):D121–D127, January 2019.

[9] Ashwini Jeggari, Debora S Marks, and Erik Larsson. mircode: a map of putative microRNA target sites in the long non-coding transcriptome. *Bioinformatics*, 28(15):2062–2063, August 2012.

[10] Jun-Hao Li, Shun Liu, Hui Zhou, Liang-Hu Qu, and Jian-Hua Yang. starbase v2.0: decoding miRNA-ceRNA, miRNA-ncRNA and protein–RNA interaction networks from large-scale CLIP-Seq data. *Nucleic Acids Res.*, 42(D1):D92–D97, November 2013.

[11] Yvonne Tay, John Rinn, and Pier Paolo Pandolfi. The multilayered complexity of ceRNA crosstalk and competition. *Nature*, 505(7483):344–352, January 2014.
